# Classification Approach for Attention Assessment via Singular Spectrum Analysis Based on Single-Channel Electroencephalograms

**DOI:** 10.3390/s23020761

**Published:** 2023-01-09

**Authors:** Weirong Wu, Bingo Wing-Kuen Ling, Ruilin Li, Zhengjia Lin, Qing Liu, Jizhen Shao, Charlotte Yuk-Fan Ho

**Affiliations:** School of Information Engineering, Guangdong University of Technology, Guangzhou 510006, China

**Keywords:** singular spectrum analysis, empirical mode decomposition, single-channel electroencephalograms, attention assessment, random forest, back-propagation neural network, support vector machine

## Abstract

Attention refers to the human psychological ability to focus on doing an activity. The attention assessment plays an important role in diagnosing attention deficit hyperactivity disorder (ADHD). In this paper, the attention assessment is performed via a classification approach. First, the single-channel electroencephalograms (EEGs) are acquired from various participants when they perform various activities. Then, fast Fourier transform (FFT) is applied to the acquired EEGs, and the high-frequency components are discarded for performing denoising. Next, empirical mode decomposition (EMD) is applied to remove the underlying trend of the signals. In order to extract more features, singular spectrum analysis (SSA) is employed to increase the total number of the components. Finally, some typical models such as the random forest-based classifier, the support vector machine (SVM)-based classifier, and the back-propagation (BP) neural network-based classifier are used for performing the classifications. Here, the percentages of the classification accuracies are employed as the attention scores. The computer numerical simulation results show that our proposed method yields a higher classification performance compared to the traditional methods without performing the EMD and SSA.

## 1. Introduction

Our brain receives a lot of sensory data generated from the environment every day. It is essential to pay attention when these data are being received in order not to miss them for further processing [1]. In fact, attention can be understood as an attribute or a feature of perceptual mechanisms and it is used to perform multiple cognitive control. Moreover, it is one of the five basic factors affecting intelligence [2], and it is used to evaluate the intellectual ability of a human.

Nowadays, both parents in major cities work outside during the daytime and leave their children at home after school. According to the survey, China has 23 million children being left at home, and the total number of children separated from their parents is increasing year by year. With the development of Internet technology, parents learn more about their children via electronic means. An attention assessment helps parents to find out the interests of their children, and plan the development of their children. Therefore, an attention assessment plays an important role in education development. On the other hand, according to the latest report conducted by the CDC in the United States, there is a child with the autism in every 44 children. In addition, the total number of autistic patients in China is more than 10 million, in which more than 3 million of the autistic patients are children. Moreover, the prevalence of ADHD in the world is as high as 6–14%. Nevertheless, severe cases of ADHD have a shorter life expectancy by 25 years. Furthermore, ADHD usually affects men more than women. More precisely, 12.9% of men and 4.9% of women are diagnosed with ADHD. In fact, the attention assessment also plays an important role in the diagnosis of ADHD and autism. This is because participants with autism highly concentrate on their work if they are interested in. On the other hand, they are highly immersed in their work if they are not interested in.

It is worth noting that different medical officers sometimes give very different attention scores to the same participant doing the same task at the same time. Hence, it is very difficult to have a subjective and reliable attention assessment. As EEGs reflect the electrophysiological activity of the neurons [3], they contain the physiological and pathological information of the human brain. In particular, the α waves are related to the attention levels when the participants work on a particular task, the β waves are related to the emotional and cognitive status of the participants [4], the θ waves are related to the moral status of the participants [5], and the δ waves are related to the attention levels when the participants perform mental tasks [6]. Hence, EEGs are widely used for the understanding of brain activities [7] and the analysis of human emotions due to personality inference. As a result, EEGs are widely used for performing sleep-stage classification, monitoring physiological state, applying intelligent rehabilitation therapy, identifying personality characteristics [8], as well as performing the diagnosis and treatment of sleep-related diseases [9] via brain-computer interface (BCI) systems [10,11]. Recently, EEGs are employed for performing automatic attention assessment [12]. In particular, the participants wear a wearable and portable device for acquiring the EEGs when they are playing an electronic game. Then, an analysis is performed based on the EEGs. An indication of the attention level is reflected in the electronic game. However, this attention assessment is designed only for participants who play electronic games [13]. Nevertheless, children are not only playing electronic games, but they also do other activities at home. Therefore, it would be useful if the attention assessment can be performed for participants doing other activities. To address this issue, this paper proposes an attention assessment method for participants doing other activities.

At present, single-channel EEGs are used for the consumer-level applications. This is because single-channel EEGs are portable and easily acquired. In addition, the costs of single-channel EEG devices are affordable to consumers. More importantly, single-channel EEGs can operate at higher sampling rates under the same data transmission rate. This will yield a better classification performance. Hence, this paper employs single-channel EEGs for performing the classification.

It is worth noting that the traditional method uses infinite impulse response filters such as the Butterworth filter to suppress noise. However, these filters introduce nonlinear phase distortion to the extracted waves. Therefore, the classification performance based on the extracted waves is degraded. To address this issue, this paper performs the FFT on the EEGs. Then, only the corresponding FFT coefficients are retained. This operation behaves similarly to idea filtering. As the retained FFT coefficients are conjugate pairs, no phase distortion is introduced. Finally, the inverse FFT is computed to obtain the denoised EEGs.

EMD is a nonlinear and adaptive signal representation method that decomposes a signal into the sum of a small number of interpretable components. It has been used for performing the underlying trend extraction. In particular, as the last several components of the EMD exhibit slowly varying oscillations, discarding these components can remove the underlying trend of the EEGs due to the motion artifacts. Hence, this paper employs the EMD for removing the underlying trend of the EEGs.

To perform the four sleep-stage classifications [14,15], the traditional methods are based on decomposing the EEGs into the β waves, α waves, θ waves, γ waves, and δ waves. Then, the features are extracted from these brain waves. However, the total number of the extracted features is very limited. As a result, the classification accuracy is low. To address this issue, it is worth noting that the SSA is also a nonlinear and adaptive signal representation method that decomposes a signal into the sum of a small number of interpretable components. Hence, this paper employs the SSA to decompose each EMD component into further components. As the total number of components is increased, more features can be extracted. In particular, this paper also extracts 11 different features from each group of SSA components. The computer numerical simulation results show that our proposed method greatly improves the classification performance. In fact, SSA has been used for processing single-channel EEGs [16] and EOGs [17], as well as for performing high-precision seizure detection and EEG classification [18].

It is worth noting that the automatic attention assessment is in fact a regression problem. The common regression methods include the logistic regression method, polynomial regression method, and ridge regression method. Nevertheless, these regression methods require subjective and reliable reference values for the attention levels. Hence, the results obtained via these regression methods are not reliable. To address this issue, this paper proposes a classification approach to perform the attention assessment. In this case, the reference attention levels are not required.

The objective of this paper is to propose a method for performing a subjective and effective attention assessment with participants conducting various activities. The novelty and contribution of this paper are to propose a classification method to perform the regression task. In this case, reference values from medical officers are not required. In addition, this paper proposes to employ SSA to increase the total number of features to improve the classification accuracy. The outline of this paper is as follows: Section 2 reviews the existing nonlinear time–frequency analysis techniques. Section 3 presents our proposed method. Section 4 shows the computer numerical simulation results. Finally, a conclusion is summarized in Section 5.

## 2. Review on the Existing Nonlinear Time–Frequency Analysis Techniques

### 2.1. SSA

SSA is a recently proposed method for performing nonlinear and adaptive signal decomposition. Since no pre-defined kernel is required [9], it is found to be very useful for analyzing signals. As a result, it is widely used in the mathematics, physics, finance, meteorology, social science, and biomedical signal processing communities.

The procedures for performing the SSA are summarized as follows: First, it constructs a trajectory matrix using the given signal. Second, singular value decomposition (SVD) is applied to the trajectory matrix, and various components, such as the underlying trend, periodic, and noise components, are obtained. Then, the useful components are retained, while the rest of the components are discarded. Finally, diagonal averaging is performed. In particular, let N be the length of a signal. Let $\;x\;={\left[x_{1},x_{2},\ldots ,x_{N}\right]}^{T}$ be the vector of this signal. Let L be the window length. Here, 1<L ≤ N/2. Define K=N − L +1. Let ${\;X}_{D}={\left[x_{D},x_{D+1},\ldots ,x_{D+L-1}\right]}^{T}$ for D=1,…,K be the Dth segment of the signal. Let X= [$X_{1}$,$X_{2}$,…,$X_{K}$] be the trajectory matrix such that the Dth column of X is $X_{D}$. That is,

$$X=\left[\begin{array}{c} x_{1}\;\;\;\;\;x_{2\;\;}\;\;\;\;\;\cdots \;\;\;x_{N-L+1}\\ x_{2}\;\;\;\;\;x_{3\;\;}\;\;\;\;\;\cdots \;\;\;x_{N-L+2}\\ \;\;\vdots \;\;\;\;\;\;\;\;\vdots \;\;\;\;\;\;\;\;\ddots \;\;\;\;\;\;\;\;\vdots \;\;\;\;\;\;\;\\ \;x_{L}\;\;\;\;x_{L+1\;\;}\;\;\cdots \;\;\;\;\;\;x_{N}\;\;\;\;\;\; \end{array}\right].$$

It is worth noting that X is a Hankel matrix. The second step for performing the SSA is to apply SVD to X to obtain the various components. Let U and V be the left unitary matrix and the right unitary matrix obtained by applying SVD to X, respectively. However, the computational complexity is very large if SVD is applied to X directly. To address this issue, SVD is applied to the covariance matrix ${\mathrm{XX}}^{T}$ to obtain U. Then, V is derived based on both U and X. Let Λ be the diagonal matrix obtained by performing SVD on ${\mathrm{XX}}^{T}$. It is worth noting that the singular values and the singular vectors of ${\mathrm{XX}}^{T}$ are the squares of the singular values and the left singular vectors of X, respectively. That is,

$${\mathrm{XX}}^{T}={\;U\Lambda U}^{T}.$$

Let $\lambda_{1},\ldots ,{\;\lambda }_{L}$ be the diagonal elements in Λ . That is, $\Lambda =\mathrm{diag}\left(\lambda_{1},\ldots ,{\;\lambda }_{L}\right)\;$. Here, $\lambda_{i}$ are sorted in descending order. That is, $\lambda_{1}\geq \cdots \geq \cdots \lambda_{L}\geq 0$. Let $U=\left[U_{1},\ldots ,{\;U}_{L}\right]$ and $V=\left[V_{1},\ldots ,{\;V}_{N-L+1}\right]$. Let

$$S_{i}=\sqrt{\lambda_{i}}U_{i}V_{i}^{T}.$$

It can be shown that X can be represented as the sum of $S_{i}$. That is,

$$X=S_{i}+\cdots +S_{L}=\sum_{i=1}^{L}\sqrt{\lambda_{i}}U_{i}V_{i}^{T}.$$

It can be shown that

$$V_{i}=\frac{X^{T}U_{i}}{\sqrt{\lambda_{i}}}\;\;for\;i=1,\ldots ,L.$$

Next, $S_{i}$ are grouped based on a certain criterion. Assume that the index set {1, 2,…, L} is partitioned into a finite number of disjoint subsets. Let M be the total number of these disjoint subsets. Let $I_{1},\ldots ,{\;I}_{M}$ be these disjointed subsets. Let ${\hat{X}}_{k}=\sum_{i\in I_{k}}S_{i}\;\mathrm{for}\;k=1,\ldots ,M$. It can be shown that X can be represented as

$$X={\hat{X}}_{1}+\cdots +{\hat{X}}_{M}.$$

**Figure 1:**
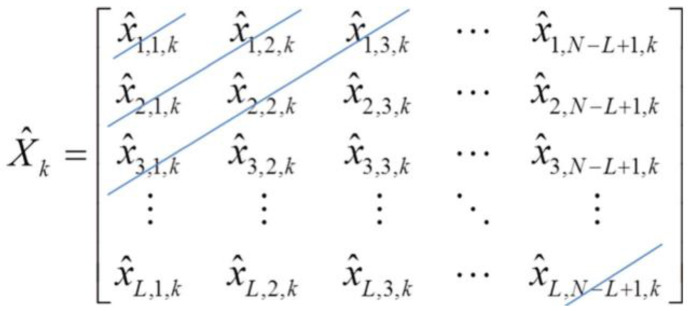
The diagonal averaging operation.

However, only some terms are summed together to construct a useful signal. The third step for performing the SSA is to perform diagonal averaging on ${\hat{X}}_{k}.$ This process involves representing ${\hat{X}}_{k}$ as a one-dimensional vector. It can be seen from (6) that the sum of the elements in the same positions of all ${\hat{X}}_{k}$ is equal to the corresponding element in the trajectory matrix. Hence, applying diagonal averaging on each ${\hat{X}}_{k}$ to obtain the corresponding one-dimensional SSA component and then summing up all these one-dimensional SSA components together is equivalent to applying diagonal averaging to the trajectory matrix. Therefore, diagonal averaging is an inverse operation with respect to the Hankelization. Let ${\hat{x}}_{a,b,k}$ be an element in the ath row and the bth column of ${\hat{X}}_{k}$. Let ${\bar{x}}_{t,k}$ be the tth element in the kth one-dimensional SSA component. Performing diagonal averaging involves computing the average of all the elements along the tth off-diagonal of ${\hat{X}}_{k}$ as shown in Figure 1. Here, t=a+b−1.

From Figure 1, we have:

$$\{\begin{array}{c} {\bar{x}}_{1,k}={\hat{x}}_{1,1,k}\\ {\bar{x}}_{2,k}=\frac{1}{2}\left({\hat{x}}_{1,2,k}+{\hat{x}}_{2,1,k}\right)\\ {\bar{x}}_{3,k}=\frac{1}{3}\left({\hat{x}}_{1,3,k}+{\hat{x}}_{2,2,k}+{\hat{x}}_{3,1,k}\right)\\ \cdots \end{array}$$

Mathematically, diagonal averaging is equivalent to performing the following operations [19,20]:

$${\bar{x}}_{t,k}=\{\begin{array}{c} \frac{1}{t}\sum_{m=1}^{t}{\hat{x}}_{m,t-m+1,\text{k}}\;\;\;\;\;\;\;\;\;\;\;\;\;\;\;\;\;\;\;\;\;\;\;\;\;\;\;\;\;\;\;\;\;\;\;\;\;\;\;\;\;\;\mathrm{for}\;1\leq t<L\\ \frac{1}{L}\sum_{m=1}^{L}{\hat{x}}_{m,\text{t}-m+1,k}\;\;\;\;\;\;\;\;\;\;\;\;\;\;\;\;\;\;\;\;\;\;\;\;\;\;\;\;\;\;\;\;\;\;\;\;\;\;\;\;\;\;\;\mathrm{for}\;L\leq t<K\\ \frac{1}{N-t+1}\sum_{m=t-K+1}^{L}{\hat{x}}_{m,t-m+1,k}\;\;\;\;\;\;\;\;\;\;\;\;\;\;\;\;\;\;\;\mathrm{for}\;K\leq t<N \end{array}.$$

Let ${\bar{x}}_{k}={\left[{\bar{x}}_{1,k},{\bar{x}}_{2,k}\ldots {\bar{x}}_{N,k}\right]}^{T}$ be the obtained one-dimensional SSA component.

### 2.2. EMD

Suppose that a signal is composed of a finite number of components. EMD is an algorithm for finding these components. More precisely, EMD is used to represent a signal as the sum of various components. These components are called intrinsic mode functions (IMFs). If the IMFs are different from the actual components, then mode mixing occurs. Here, the EMD does not require any pre-defined kernel for performing the decomposition. As the IMFs are only dependent on the signal itself, EMD is an adaptive signal decomposition method. On the other hand, it is worth noting that the decomposition is based on the time-scale characteristics of the given signal. As a signal with more extrema contains more high-frequency components than a signal with less extrema, EMD exploits the time–frequency characteristics of the signal. These excellent properties of EMD facilitate many science and engineering applications [21,22,23].

A function is said to be an IMF if it satisfies the following two conditions:

(1) The absolute difference between the total number of extrema and the total number of zero-crossing points is no more than 1.
(2) The area under the curve between two consecutive extrema is zero.

The procedures for performing the EMD are as follows:

Step 1 Initialization: Let $r_{0}\left(t\right)=x\left(t\right)$ and n=1.
Step 2 Compute the IMFs: Let $c_{n}$(t) be the nth IMF. It is found using the following procedures:
  (a) Let $d_{0}\left(t\right)=r_{n-1}\left(t\right)$ and j=1.
  (b) Find the maxima and minima of $d_{j-1}\left(t\right)$.
  (c) The cubic spline function is used to interpolate among the maxima and minima to obtain the upper and lower envelope of $d_{j-1}\left(t\right)$, respectively. Let $e_{\max}\left(t\right)$ and $e_{\min}\left(t\right)$ be the upper and lower envelope of $d_{j-1}\left(t\right)$, respectively.
  (d) Calculate the mean of $e_{\max}\left(t\right)$ and $e_{\min}\left(t\right)$. Let m(t) be this mean. That is, $m\left(t\right)=\frac{e_{max}\left(t\right)+e_{min}\left(t\right)}{2}$.
  (e) Performing the sifting process via subtracting m(t) from $d_{j-1}\left(t\right)$. That is, $d_{j}\left(t\right)=d_{j-1}\left(t\right)-m\left(t\right)$.
  (f) A criterion is imposed to terminate the algorithm. Let $\mathrm{SD}=\sum_{t}\frac{{\left|m\left(t\right)\right|}^{2}}{{\left|d_{j-1}\left(t\right)\right|}^{2}}$. If SD<0.3, then let $c_{n}\left(t\right)=d_{j}\left(t\right)$ and go to Step 3. Otherwise, increment the value of j and go back to Step 2b.
Step 3 Let $r_{n}\left(t\right)=r_{n-1}\left(t\right)-c_{n}\left(t\right)$.
Step 4 If $r_{n}\left(t\right)$ is an IMF or a monotonic function, then the decomposition is completed. Otherwise, increment the value of n and go back to Step 2a.

Let N−1 be the total number of the obtained IMFs and $r_{N-1}\left(t\right)$ be the residue. Then, x(t) can be represented as the sum of these N−1 IMFs and the monotonic residue as follow:

$$x\left(t\right)=\sum_{i=1}^{N-1}c_{i}\left(t\right)+r_{N-1}\left(t\right).$$

For simplicity reasons, let $c_{N}\left(t\right)=r_{N-1}\left(t\right)$.

## 3. Our Proposed Method

**Figure 2:**
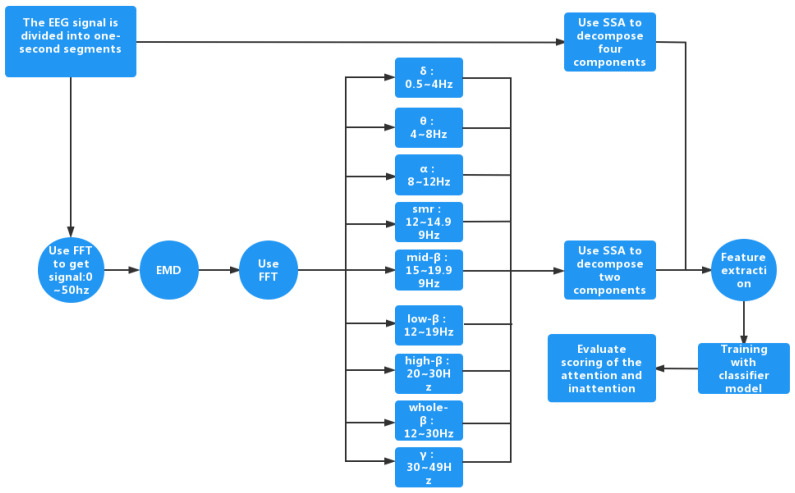
The block diagram of our proposed method.

Figure 2 shows the block diagram of our proposed method. The main idea of our proposed method is as follows: First, in order to perform the attention assessment, two sets of EEGs are acquired. They are the sets of EEGs where the participants perform different activities under the concentration state and the immersion state. Second, since the useful information of the EEGs is localized between 0 Hz and 50 Hz and the components outside 50 Hz can be understood as noise, FFT is used to perform the denoising. In particular, the FFT coefficients between 0 Hz and 50 Hz are retained and those outside 50 Hz are discarded. Third, EMD is used to perform the detrending. Fourth, FFT is applied again to obtain the various types of the brain waves. In particular, nine brain waves are obtained. Fifth, SSA is applied to decompose each brain wave and the detrended EEG into the various SSA components. Then, the grouping is performed. Overall, there are 22 grouped SSA components for each detrended EEG. Sixth, the features are extracted from each SSA grouped component. Seventh, the BP neural network-based classifier, random forest based-classifier, and SVM-based classifier are used to perform the training. Finally, the class of each test EEG is estimated. Due to randomness, the above operations are performed three times. The accuracy based on the average of these three classification results is taken as the final result.

### 3.1. Data Acquisition

It is worth noting that no database for performing the activity recognition using single-channel EEGs operated at 512 Hz can be found on the Internet. Hence, it is difficult to perform a fair comparison using the data in the public domain. Therefore, this paper builds a new database and the data is available on the Internet at this link: https://www.aliyundrive.com/s/Qhuc1ZFRkLr, accessed on 18 December 2022. In particular, the EEGs are acquired from 12 participants, including 9 boys and 3 girls. There were 9 participants from Guangzhou and 3 participants from Hong Kong. All of them have conducted the body test and they are certificated with a good health status. Each participant conducts seven activities. These seven activities are drawing, eating, doing computer exercises, playing electronic games, reading, playing toys, and watching television. Each activity is conducted twice with each time lasting for around 10 to 15 min. Here, each activity conducted at the first time and the second time are under the concentration state and the immersion state, respectively. For conducting the activities under the concentration state, there is no interruption to the participants. In addition, the surrounding environment is particularly quiet without any noise so that the participants can easily focus on the activities they performed. Moreover, since it is difficult for the participants to focus on the activities for a long period of time, different activities conducted by the individual participants are carried out on different days. Here, it is assumed that the attentions of the participants are independent of the day when they perform the activities. For conducting the activities under the immersion state, the participants are disturbed when they conduct these activities. For example, the ears of the participants are disrupted by feathers or they are talking to one another all the time.

**Figure 3:**
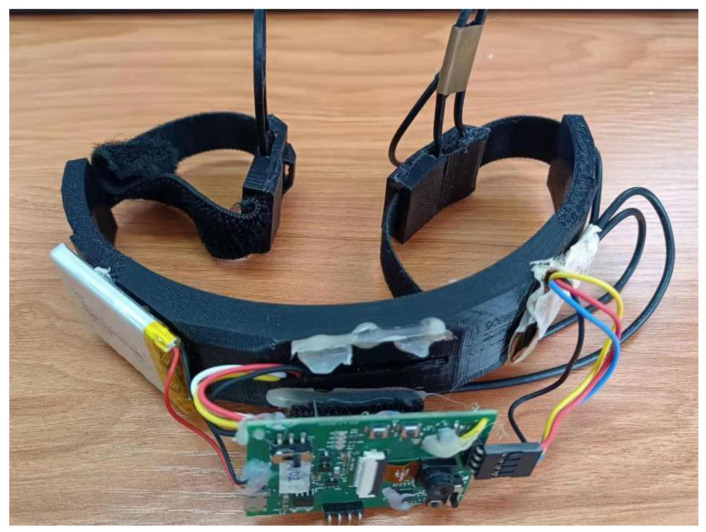
The acquisition device.

Moreover, the NeuroSky TGAM1 chip is used as the sensor to acquire the single-channel EEGs with the sampling frequency operating at 512 Hz. Figure 3 shows the acquisition device. This EEG device has three contact points. The sensor is placed at the TP10 location near the right ear lobe because there is no bone there. Hence, the EEGs can be sensed by the electrode more easily. On the other hand, the other two contact points are the reference point (REF) and the ground point (GND). They are located at the forehead because the contact surface is large there. Hence, it can avoid poor contact. WiFi is used to transmit the EEGs acquired from this device to the xampp server built in the computer. The EEGs are segmented into pieces using an ideal rectangular window. Each piece of the EEGs lasted for one second. Hence, each piece of the EEGs consists of 512 points. Here, each piece of the EEGs is regarded as an individual sample. Within one second, it is assumed that the attention level of the participants remains unchanged. In this paper, the pieces of the EEGs acquired under the poor contact condition are removed. Table 1 and Table 2 show the total numbers of the retained samples when different participants are conducting different activities under the concentration state and the immersion state, respectively. It is found that about 13 to 806 pieces of the EEGs are retained.

### 3.2. FFT-Based Denoising

**Figure 4:**
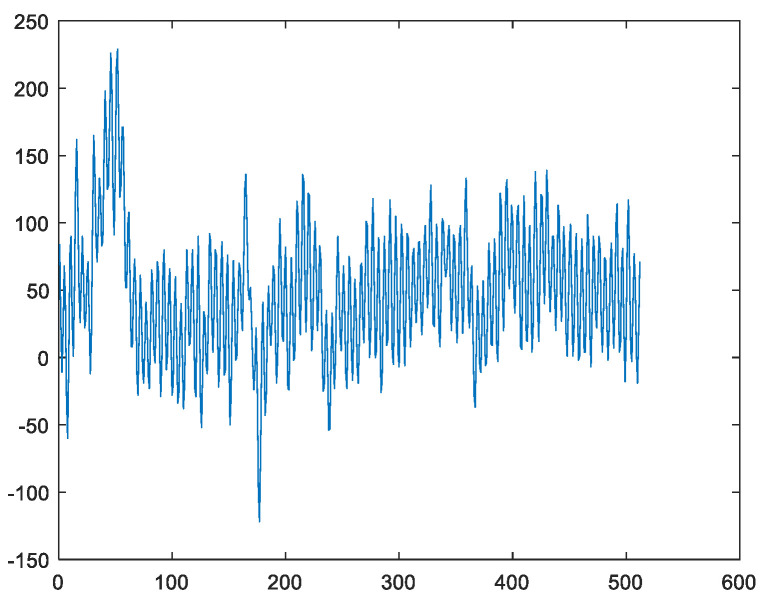
An example of a sample of the EEG when Danny is conducting the drawing activity under the concentration state.

Figure 4 shows an example of a sample of the EEG when Danny is conducting the drawing activity under the concentration state. It is well known in the EEG community that the useful information of the EEG is localized in the frequency band between 0.5 Hz and 49 Hz. However, it can be seen from Figure 4 that there is a high-frequency noise above 50 Hz contaminating the EEG. Hence, it is required to remove this high-frequency noise. Although different IMFs are localized in different frequency bands, the IMFs have leakages in the frequency domain because these IMFs are not bandlimited. Moreover, as the mode-mixing phenomenon occurs [21,23], the obtained IMFs are not the corresponding brain waves. To address this issue, the FFT approach is applied. That is, the FFT is applied to the EEG and those coefficients higher than 50 Hz are set to zero. As the remained coefficients are bandlimited, this is equivalent to applying the ideal lowpass filtering to the EEG. In this case, the high-frequency artifacts are eliminated. Moreover, the corresponding brain waves can be extracted directly via the FFT approach.

### 3.3. Removing the Underlying Trend via the EMD

**Figure 5:**
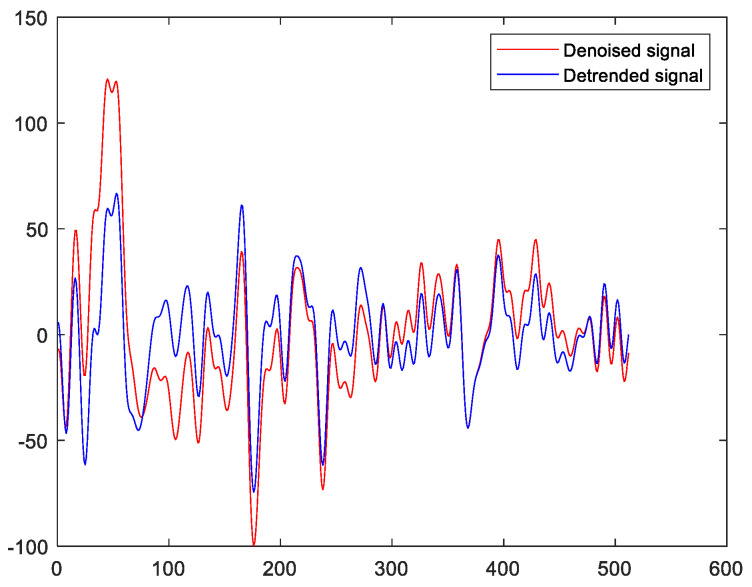
The denoised signal and the detrended signal.

It is worth noting that the eye and muscular activities would introduce drift to the acquired EEGs. In fact, this drift can be characterized by the underlying trend of the EEGs. On the other hand, the EMD represents a signal as the sum of its IMFs with different IMFs localized in different frequency bands. It is well known that the underlying trend of a signal can be characterized by the sum of its last several IMFs. This is because the last several IMFs usually have large time scales and they are also low-frequency components. These properties are the same as those of the underlying trend [24]. To address this issue, this paper applies EMD to the denoised EEGs obtained in Section 3.2, and the last several IMFs corresponding to the underlying trend components and the low-frequency components of the EEGs are discarded. As a result, the effects of the eye and muscular activities are eliminated. More precisely, this paper removes the last two IMFs. Figure 5 shows the denoised EEG and the corresponding detrended EEG. It can be seen from Figure 5 that the average value of the denoised signal is not equal to zero. This implies that there is an underlying trend in the denoised signal. On the other hand, this is not the case for the detrended signal. This implies that the underlying trend of the denoised signal is effectively removed via the EMD approach.

### 3.4. Feature Extraction Based on the SSA

**Figure 6:**
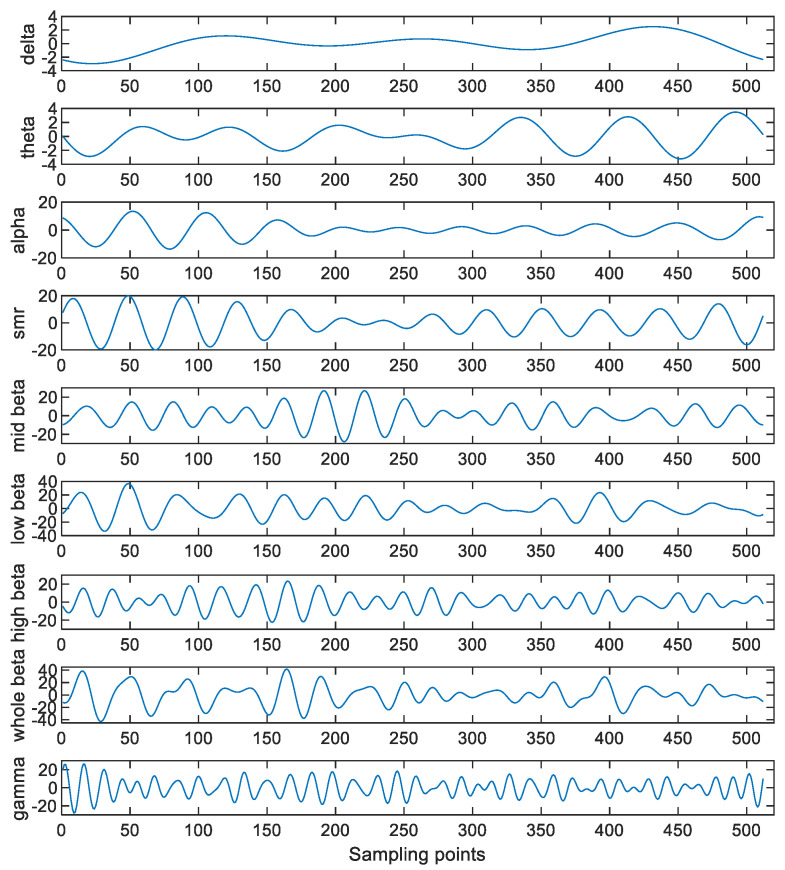
The obtained brain waves when Danny is conducting the drawing activity under the concentration state.

The EEGs consist of the various brain waves localized in different frequency bands. The frequency band of the δ wave is between 0.5 Hz and 4 Hz, that of the θ wave is between 4 Hz and 8 Hz, that of the α wave is between 8 Hz and 12 Hz, that of the sensory motor rhythm (SMR) wave is between 12 Hz and 14.99 Hz, that of the mid β wave is between 15 Hz and 19.99 Hz, that of the high β wave is between 20 Hz and 30 Hz, that of the low β wave is between 12 Hz and 19 Hz, that of the whole β wave is between 12 Hz and 30 Hz, and that of the γ wave is between 30 Hz and 49 Hz. To extract these waves from the EEG, an FFT approach is applied to the detrended EEGs obtained in Section 3.3. That is, the FFT is applied to the EEGs and the coefficients outside the corresponding frequency bands are set to zero. Then, the inverse FFT (IFFT) is applied to the processed signals to obtain the corresponding waves. Figure 6 shows the obtained brain waves when Danny is conducting the drawing activity under the concentration state.

It is worth noting that the FFT has been applied twice as discussed in Section 3.2 and Section 3.4, the first time to perform the denoising, the second time to extract the various waves from the denoised and the detrended EEGs. In fact, these two FFT operations cannot be combined. This is because the duration of the samples is one second. Hence, the frequency resolution of the FFT coefficients is 1 Hz. Even when the DC frequency of the samples is set to zero, the detrended performance is not good. Therefore, EMD is required to be employed to remove the underlying trend of the EEGs, where the detrended operation is performed between these two FFT operations.

**Figure 7:**
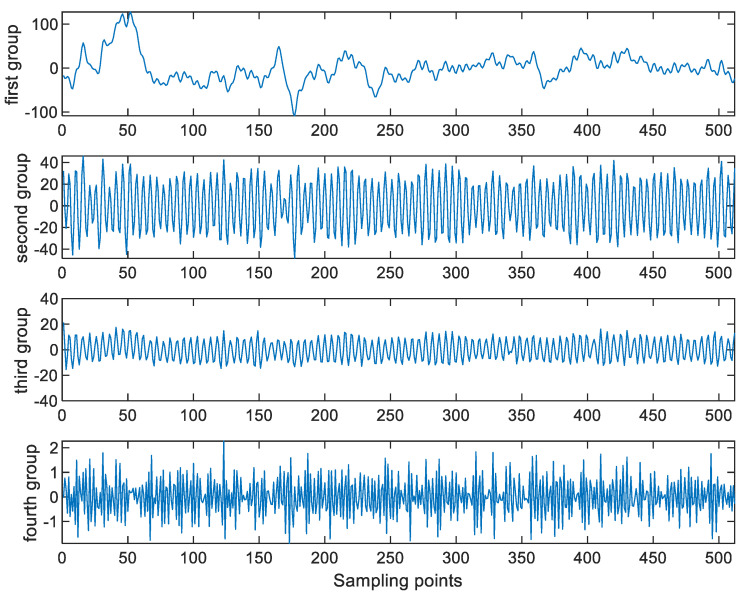
The four groups of the SSA components.

**Figure 8:**
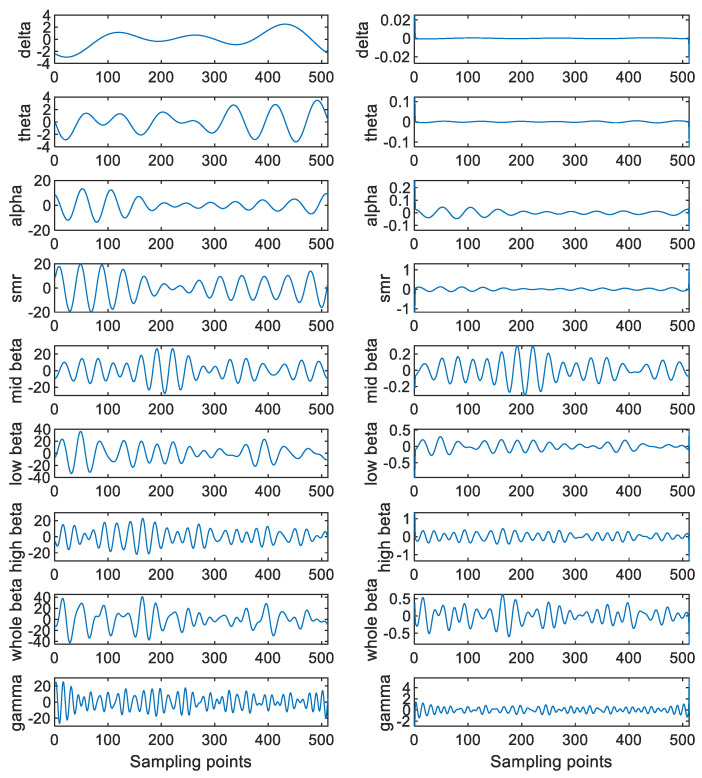
These two sets of the SSA components decomposed from the brain waves of a detrended EEG.

Table 3 shows some common human behaviors and the brain waves responsible for these human behaviors. As the brain waves are related to the human behaviors, this paper extracts the features from these brain waves for performing the attention assessment. On the other hand, it is worth noting that more than one component can be obtained via performing the SSA. Hence, more features can be extracted by applying the SSA to these brain waves. In particular, the SSA is applied to both the detrended EEGs and the decomposed brain waves. Here, L is set at 6 for the detrended EEGs, so there are six SSA components for each detrended EEG. Then, these six SSA components are categorized into four groups. More precisely, the first component is put into the first group. The second and third components are put into the second group. The fourth and fifth components are put into the third group. Finally, the sixth component is put into the fourth group. Figure 7 shows these four groups of the SSA components. Moreover, L is set at 2 for these decomposed brain waves. Figure 8 shows these two sets of the SSA components decomposed from the brain waves. Since there are four groups of the SSA components decomposed from each detrended EEG as well as two sets of the SSA components decomposed from each brain wave and there are nine brain waves for each detrended EEG, there are 22 components in total for each detrended EEG.

Since the amplitudes of different brain waves are different, the amplitudes and energies of the SSA components are not used as features for performing the attention assessment. Instead, this paper employs other physical quantities such as the approximate entropy, the mean, the variance, the interquartile range, the mean absolute deviation, the range, the skewness, the kurtosis, the $L_{1}$ norm, the $L_{2}$ norm, and the $L_{\infty }$ norm of the SSA components of each detrended EEG as the features. The details of these physical quantities are presented below.

#### 3.4.1. Approximate Entropy

The approximate entropy is a physical quantity that is robust to the noise and the interference. Hence, it has been used to estimate the characteristics of the distribution of both a short random signal and a short deterministic signal acquired at both the noise environment and the interference environment [25]. On the other hand, as the EEGs behave like a random signal, the approximate entropy should be effective for analyzing the EEGs. However, it has not been used for performing the attention assessment yet. Therefore, this paper explores the feasibility and effectiveness of using the approximate entropy for performing the attention assessment. Let x(t) be an SSA component. Let m be the length of the marginal window of the SSA. Then, the SSA component is divided into a finite number of sub-sequences. Let X(i) be the ith sub-sequence. That is, $X\left(i\right)={\left[x\left(i\right),x\left(i+1\right),x\left(i+2\right),\ldots ,x\left(i+m-1\right)\right]}^{T}$. Let N be the length of this SSA component. Let K be the total number of sub-sequences. Obviously, K=N−m+1 . Let $d_{\mathrm{ij}}$ be the distance between X(i) and X(j). Here, $d_{\mathrm{ij}}$ is defined as the maximum absolute difference between X(i) and X(j). Let r be a value between 0.25 and 0.75. In this paper, r is chosen as 0.5. Let SD be the standard deviation of the SSA component. Let P be a threshold. Then, P is selected as P=r×SD. For each i, there are K distances. Let $C_{i}^{m}\left(P\right)$ be the percentage of the total number of distances having values greater than P. Let $\varphi_{m}\left(P\right)=\frac{1}{N-m+1}\sum_{i=1}^{N-m+1}\log(C_{i}^{m}\left(P\right))$ be the logarithmic average of $C_{i}^{m}\left(P\right)$. Finally, increment the value of m and repeat the above procedures. Then, we have $\varphi_{m+1}\left(P\right)$. Define $\mathrm{ApEn}=\varphi_{m}\left(P\right)-\varphi_{m+1}\left(P\right)$ as the approximate entropy.

#### 3.4.2. Mean and Variance

Let $\bar{X}=\frac{\sum_{i=1}^{N}x\left(i\right)}{N}$ be the mean of x(t).

Let $s^{2}=\frac{\sum_{i=1}^{N}{\left(x\left(i\right)-\;\bar{X}\right)}^{2}}{N-1}$ be the variance of x(t).

#### 3.4.3. Interquartile Range

Let $\tilde{x}\left(n\right)$ be the ordered EEG sorted in ascending order. Let Q1, Q2, and Q3 be the lower quartile, the median, and the upper quartile of $\tilde{x}\left(n\right)$, respectively. That is, they are the 25th percentile, the 50th percentile, and the 75th percentile of $\tilde{x}\left(n\right)$, respectively. Let IQR = Q3 − Q1.

#### 3.4.4. Mean Absolute Deviation

Let $M_{d}=\frac{\sum_{i=1}^{N}\left|x\left(i\right)-\bar{X}\right|}{N}$ be the mean absolute deviation of x(n).

#### 3.4.5. Range

Let R=xnmax(n)−xnmin(n) be the range of x(n).

#### 3.4.6. Skewness and Kurtosis

Let $\mathrm{Skew}\left(X\right)=\frac{\sum_{i=1}^{N}{\left(\frac{x\left(i\right)-\;\bar{X}}{s}\right)}^{3}}{N}$ be the skewness of x(n).

Let $\mathrm{Kurt}\left(X\right)=\frac{\sum_{i=1}^{N}{\left(\frac{x\left(i\right)-\;\bar{X}}{s}\right)}^{4}}{N}$ be the kurtosis of x(n).

#### 3.4.7. L_1_ Norm, $L_{2}$ Norm, and $L_{\infty }$ Norm

Let ${\left|\left|X\left(t\right)\right|\right|}_{1}=\sum_{n=1}^{N}\left|x\left(n\right)\right|$ be the $L_{1}$ norm of x(n).

Let ${\left|\left|X\left(t\right)\right|\right|}_{2}=\sqrt{\sum_{n=1}^{N}{\left|x\left(n\right)\right|}^{2}}$ be the $L_{2}$ norm of x(n).

Let ||X(t)||∞=|x(n)|nmax be the $L_{\infty }$ norm of x(n).

It is worth noting that there are 11 features for each SSA component. As there are 22 components for each detrended EEG, the length of the feature vector is 242.

In order to verify whether these features are effective for performing the attention assessment, the ANOVA is performed on these features. The implementation was conducted using the Matlab statistical toolbox with the confidence level set at 95%. Let P be the probability of producing a statistical significance. Then, the power of the hypothesis testing refers to the percentage of the cases where P is smaller than one minus the confidence level. In this case, if P is less than 0.05, then these features are statistically significant. The hypothesis test results are shown in Table 4. It can be seen from Table 4 that many values of P are less than 0.05. In particular, it can be seen from the Table 4 that 195 values of P were less than 0.05. Hence, the power of the hypothesis testing was 80.58%. It can be concluded that these features are effective.

### 3.5. Classification

It is challenging to assess human attention via EEGs. This is because there is no standard method to score the degree of attention of the participants when they conduct the various activities. To address this issue, this paper employs the features extracted from the EEGs acquired from the various participants conducting the various activities under both the concentration state and the immersion state to train the classifiers. Here, three common classifiers, namely the random forest-based classifier, the SVM-based classifier, and the BP neural network-based classifier are employed for performing the classification. However, the total number of EEGs for a particular activity corresponding to the concentration state is different from that corresponding to the immersion state. Hence, there is a data imbalance issue. To address this issue, this paper employs 30% of all the EEGs for each activity for performing the testing. For the remaining 70% of all the EEGs for each activity, it first forms a preliminary training set. Then, only a part of the EEGs in this preliminary training set is taken out to construct the model for performing the classification. In particular, the total number of EEGs for each activity corresponding to the concentration state used for performing the training is taken to be the same as that corresponding to the immersion state. After performing the classification, the percentage of the total number of the EEGs that are classified correctly is computed. Here, it is assumed that the classification error is only due to the change of the state when the participant conducts the corresponding activity. Therefore, this percentage can be used to score human attention.

#### 3.5.1. Random Forest-Based Classifier

**Figure 9:**
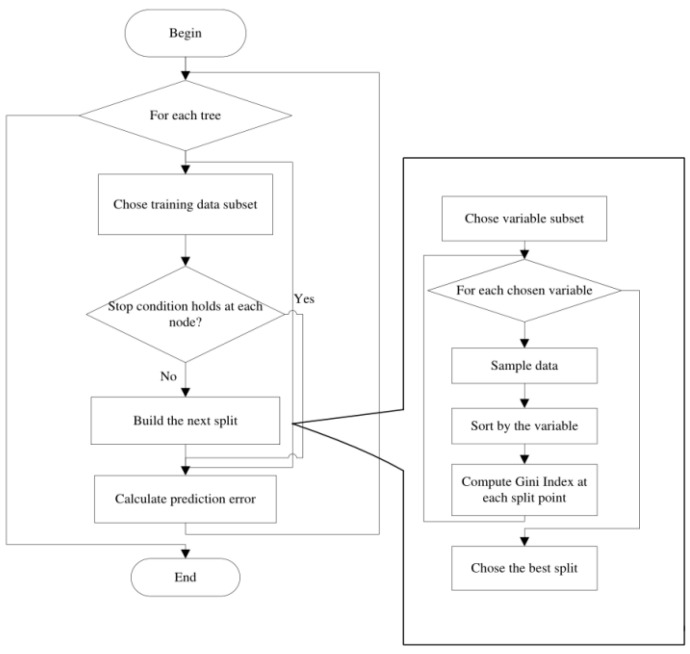
The procedures for the formation of the random forest.

The random forest is a bagging and a decision tree-based machine learning method [26]. Assume that there are N samples in the training set. The bootstrap method is used to select a sample at each iteration from these N samples randomly. To determine the splitting nodes in the decision tree, some features are selected randomly. The Gini index is computed to determine the optimal splitting node. These procedures are repeated until it cannot be further split. Finally, the above procedures are repeated and these decision trees form a random forest. Figure 9 shows the process for the formation of the random forest.

A decision tree in the random forest is a weak learner. It makes an individual decision. The overall decision is made by voting the majority decision among all the individual decisions. Since the samples are selected randomly and the features in each sample are also selected randomly, this approach can raise the generalization ability of the model.

#### 3.5.2. BP Neural Network-Based Classifier

**Figure 10:**
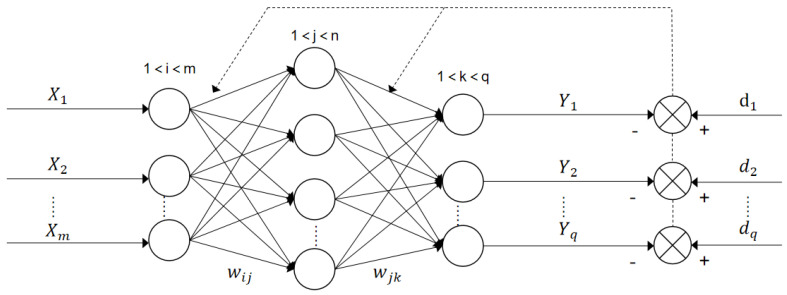
The BP neural network.

A multi-layer feedforward neural network consists of one input layer, one or more than one implicit layer, and one output layer. The implicit layer is also called the hidden layer. Training the neural network is finding the parameters in the neural network. The BP neural network is a neural network with its parameter trained by the error reverse propagation algorithm. In particular, the training composes of two major steps. The first step is the forward propagation of the signal and the second step is the BP of the error. For the first step, the input at the input layer is processed layer by layer and yields the output at the output layer. For the second step, if the output at the output layer does not match the desired output, then the error is back-propagated to the input. That is, the output is transmitted from the output layer back to the input layer through the hidden layer based on the error between the desirable output and the actual output. Here, the error is shared to all the nodes in all the layers. The weights and the thresholds of the nodes are updated via the fastest descent-based learning rule so that the total error in all the nodes is minimized. Figure 10 shows the diagram of the BP neural network [27].

#### 3.5.3. SVM-Based Classifier

As the SVM is a powerful classification method, it has been widely used in many real-world pattern recognition and the machine learning applications [28]. The SVM maps the input feature vectors in a high dimensional space to the feature vectors in a low dimensional space via a nonlinear kernel function. As the dimension of the output of the kernel function is reduced, the required computational power for computing the product of the outputs of two kernel functions ($k\left(x_{i},x_{j}\right)=\varphi \left(x_{i}\right)\varphi \left(x_{j}\right)$) is lower than that for the inner product between the weight matrix and the feaure vector. Hence, the SVM can significantly reduce the required computational power. After performing the nonlinear mapping, the linear classification method is applied in the low dimensional space. In general, the linearly separable recognition problem can be classified correctly using a linear hyperplane. To find this optimal linear hyperplane, the $L_{\infty }$ optimization approach is employed. That is, the maximum distance between the feature vectors and the linear hyperplane is minimized. However, the feasible set of the optimization problem is empty if the recognition problem is nonlinearly separable. To address this issue, the penalty factor is introduced to modify the the loss function and the classification interval. Nevertheless, the classification performance of the SVM is highly dependent on the choice of the kernel function and the error penalty factor.

## 4. Computer Numerical Simulation Results

Table 5 and Table 6 show the percentages of the classification accuracies for 12 participants performing the various activities under the concentration state and the immersion state via the traditional methods, respectively. Here, the traditional methods refer to the methods of performing both the FFT-based denoising and the classifications via the above three classifiers but without applying the SSA and the EMD to the denoised EEGs. On the other hand, Table 7 and Table 8 show the percentages of the classification accuracies for these 12 participants performing the various activities under the concentration state and the immersion state via our proposed method, respectively. Here, our proposed method refers to the method of performing the FFT-based denoising, applying both the SSA and the EMD to the denoised EEGs, as well as performing the classifications via these three classifiers. Moreover, the average percentages of the classification accuracies over both these 12 participants and over these three classifiers for each activity under the concentration state and the immersion state yielded by the traditional methods and our proposed method are shown in Table 5, Table 6, Table 7 and Table 8. It can be seen from these tables that the average percentages of the classification accuracies over both these 12 participants and over these three classifiers yielded by our proposed method are higher than those yielded by the traditional methods for all the activities as well as for both the concentration state and the immersion state. Furthermore, it is found that the highest score recorded in the concentration state yielded by our method is 100. This demonstrates that our proposed method yields a better classification performance.

Moreover, for the activities conducted in the concentration states by our proposed method, the eating activity has the lowest average score which is 68.63, the playing toy activity has the second lowest average score which is 74.24, the drawing activity has the third lowest average score which is 83.66, the playing electronic game activity has the intermediate average score which is 86.26, the watching television activity has the third highest average score which is 86.61, the doing computer exercise activity has the second highest average score which is 88.60, and the reading activity has the highest average score which is 88.75. For the activities conducted in the concentration states by the traditional method, the eating activity has the lowest average score which is 68.07, the playing toy activity has the second lowest average score which is 69.53, the drawing activity has the third lowest average score which is 74.30, the playing electronic game activity has the intermediate average score which is 80.35, the watching television activity has the third highest average score which is 80.67, the doing computer exercise activity has the second highest average score which is 82.26, and the reading activity has the highest average score which is 82.37. For the activities conducted in the immersion states by our proposed method, the eating activity has the lowest average score which is 72.33, the playing toy activity has the second lowest average score which is 76.97, the watching television activity has the third lowest average score which is 86.80, the drawing activity has the intermediate average score which is 86.96, the playing electronic game activity has the third highest average score which is 88.04, the doing computer exercise activity has the second highest average score which is 88.05, and the reading activity has the highest average score which is 91.34. Here, it can be seen that there are the similar sorting orders for the activities yielded by both our proposed method and the traditional methods for both the concentration state and the immersion state. This demonstrates that our proposed method is reliable. Moreover, as it is required to pay more attention when performing the reading activity and the doing computer exercise activity compared to performing the eating activity and the playing toy activity, the obtained results are reasonable.

Furthermore, it is worth noting that it is not necessarily true for the average percentages of the classification accuracies over both these 12 participants and over these three classifiers for performing the various activities yielded by both our proposed method and the traditional methods under the concentration state to be higher than those under the immersion state.

**Figure 11:**
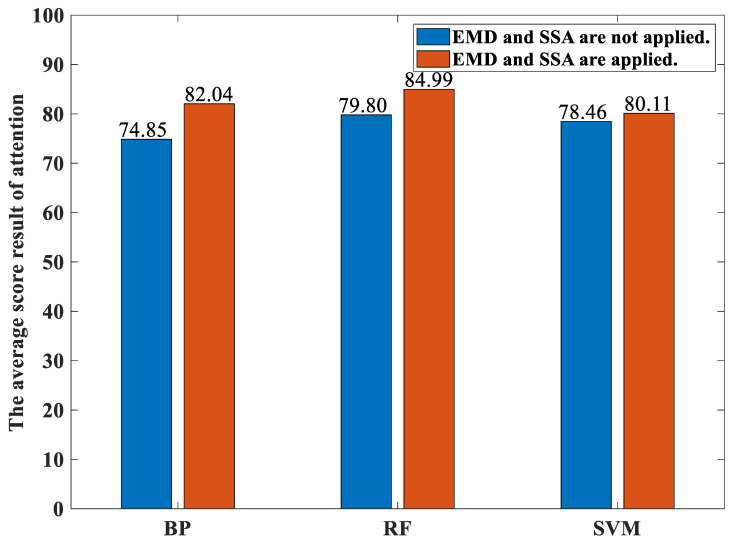
The average percentages of the classification accuracies over both these 12 participants performing the various activities yielded by both our proposed method and the traditional methods with different classifiers under the concentration state.

**Figure 12:**
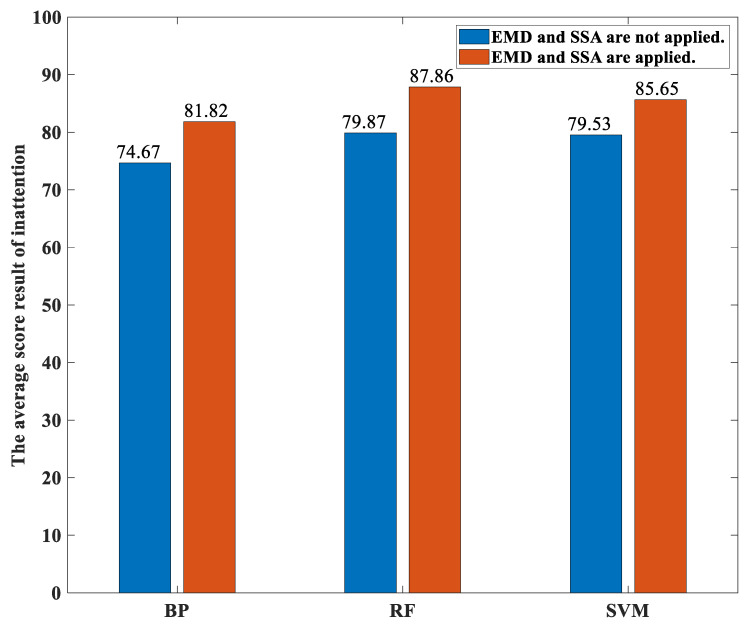
The average percentages of the classification accuracies over both these 12 participants performing the various activities yielded by both our proposed method and the traditional methods with different classifiers under the immersion state.

Figure 11 shows the average percentages of the classification accuracies over both these 12 participants performing the various activities yielded by both our proposed method and the traditional methods with different classifiers under the concentration state. Likewise, Figure 12 shows the average percentages of the classification accuracies over both these 12 participants performing the various activities yielded by both our proposed method and the traditional methods with different classifiers under the immersion state. For the concentration state, the average percentages of the classification accuracies over both these 12 participants performing the various activities yielded by our proposed method based on the the BP neural network-based classifier, the random forest-based classifier, and the SVM-based classifier models are 82.04, 84.99, and 80.11, respectively. The average percentages of the classification accuracies over both these 12 participants performing the various activities yielded by the traditional method based on the the BP neural network-based classifier, the random forest-based classifier, and the SVM-based classifier models are 74.85, 79.80, and 78.46, respectively. For the immersion state, the average percentages of the classification accuracies over both these 12 participants performing the various activities yielded by our proposed method based on the the BP neural network-based classifier, the random forest-based classifier, and the SVM-based classifier models are 81.82, 87.86, and 85.65, respectively. The average percentages of the classification accuracies over both these 12 participants performing the various activities yielded by the traditional method based on the BP neural network-based classifier, the random forest-based classifier, and the SVM-based classifier models are 74.67, 79.87, and 79.53, respectively. It can be seen from these two figures that the random forest-based classifier yields the highest average percentages of the classification accuracies compared to the SVM-based classifier and the BP neural network-based classifier for both the concentration state and the immersion state as well as for both our proposed method and the traditional method. This is because the random forest introduces randomness in the selection of both the feature vectors and the features in the generation of the forest. Thus, the effect of the overfitting is small.

It is worth noting that the duration of each sample shown in the above computer numerical simulations is one second. It is interesting to see if the percentages of the classification accuracies would depend on the duration of the samples. Now, the duration of each sample is changed to three seconds, five seconds, and ten seconds. The percentages of the classification accuracies yielded by our proposed method via the various classifiers based on the EEGs at different durations of the samples acquired from Hejian performing the reading activity under the concentration state are shown in Table 9. It can be seen from Table 9 that the percentages of the classification accuracies are almost the same. In addition, the percentages of the classification accuracies based on the duration of the samples lasting for one second are the highest compared to the other durations of the samples for both the random forest-based classifier and the SVM-based classifier. Moreover, as some of the existing works are also based on the EEGs with the duration of the samples being equal to one second [29,30], this paper chooses the duration of the samples as one second.

Moreover, the total time taken required for performing the attention assessments for each individual for performing each activity under both the concentration state and the immersion state via the traditional methods with all three classifiers is shown in Table 10. Likewise, the total time taken required for performing the attention assessments for each individual for performing each activity under both the concentration state and the immersion state via our proposed method with all three classifiers is shown Table 11. Although our proposed method takes a longer time for performing the attention assessments compared to the traditional methods due to the time required for performing the extra operations on the SSA and the EMD, the extra time is affordable for medical applications.

**Figure 13:**
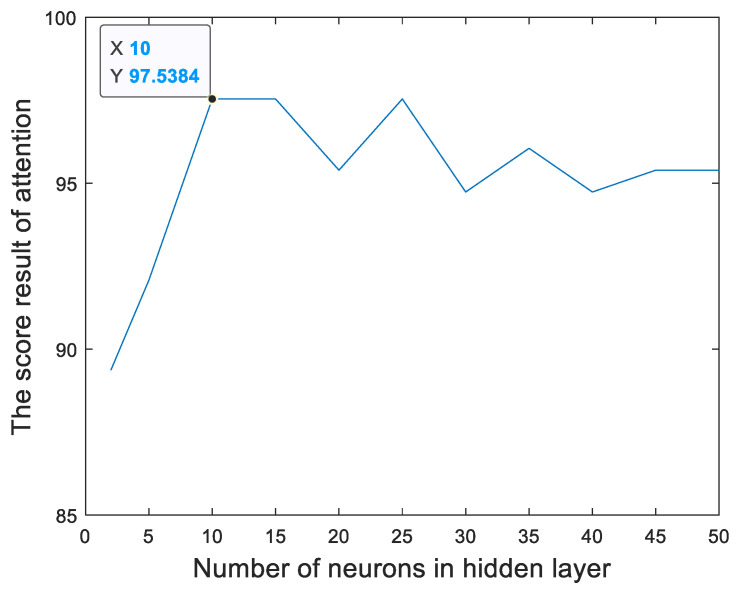
The relationship between the percentage of the classification accuracy and the total number of neurons in the hidden layer of the BP neural network in our proposed method trained using the EEGs acquired from Hejian performing the reading activity under the concentration state on the first day.

**Figure 14:**
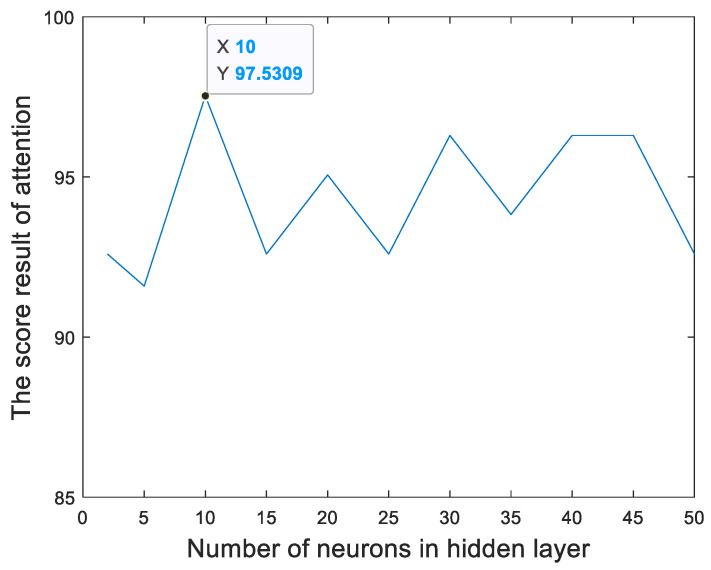
The relationship between the percentage of the classification accuracy and the total number of neurons in the hidden layer of the BP neural network in our proposed method trained using the EEGs acquired from Hejian performing the reading activity under the concentration state on the second day.

Figure 13 and Figure 14 show the relationships between the percentage of the classification accuracy and the total number of neurons in the hidden layer of the BP neural network in our proposed method trained using the EEGs acquired from Hejian performing the reading activity under the concentration state on the first day and on the second day, respectively. It can be seen that the highest percentage of the classification accuracy is yielded when 10 neurons are used in the hidden layer of the BP neural network regardless of whether the EEGs are acquired on the first day or on the second day. Hence, this paper employs 10 neurons in the hidden layer of the BP neural network.

**Figure 15:**
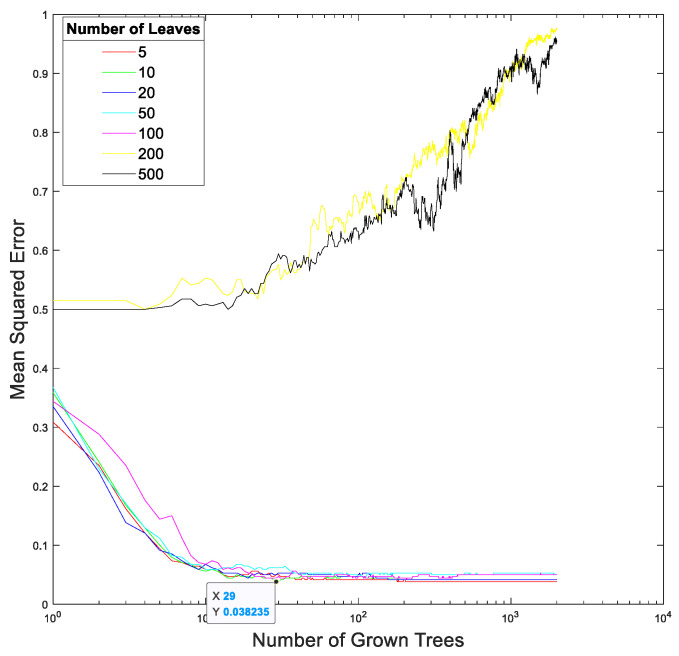
The relationship between the mean squares error and the total number of trees at different total numbers of leaf nodes in the random forest in our proposed method trained using the EEGs acquired from Hejian performing the reading activity under the concentration state on the first day.

**Figure 16:**
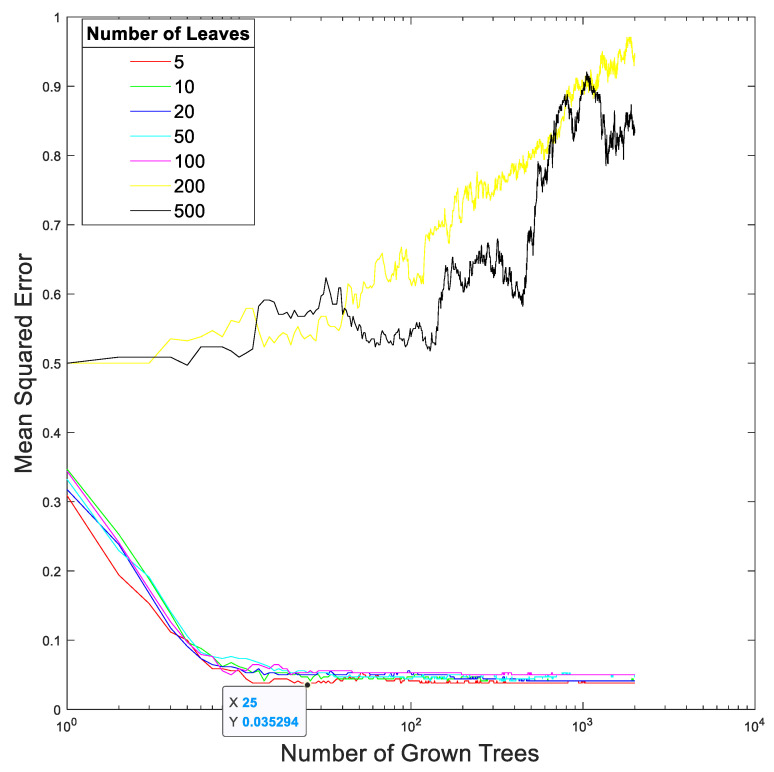
The relationship between the mean squares error and the total number of trees at different total numbers of leaf nodes in the random forest in our proposed method trained using the EEGs acquired from Hejian performing the reading activity under the concentration state on the second day.

Figure 15 and Figure 16 show the relationships between the mean squares errors and the total number of trees at different total numbers of leaf nodes in the random forest in our proposed method trained using the EEGs acquired from Hejian performing the reading activity under the concentration state on the first day and on the second day, respectively. It can be seen that the lowest mean squares errors are achieved by using 10 leaf nodes and 29 trees for the EEGs acquired on the first day as well as by using 5 leaf nodes and 25 trees for the EEGs acquired on the second day. However, the mean squares errors are insensitive to both the total number of leaf nodes and the total number of trees used in the random forest if the total number of leaf nodes is less than 100 and the total number of trees is more than 10. This is true for the EEGs acquired on both the first day and the second day. Hence, this paper selects 5 leaf nodes and 30 trees in the random forest for performing the classification.

Table 12 shows the optimal parameters in the SVM in our proposed method trained using the EEGs acquired from Hejian performing the reading activity under the concentration state on both the first day and the second day. Here, the parameters to be investigated are the value of the box constraint, the type of the kernel functions, and the parameter in the kernel function. It can be seen from Table 11 that the optimal parameters in the SVM in our proposed method trained using the EEGs acquired on the first day are very different from those acquired on the second day.

**Figure 17:**
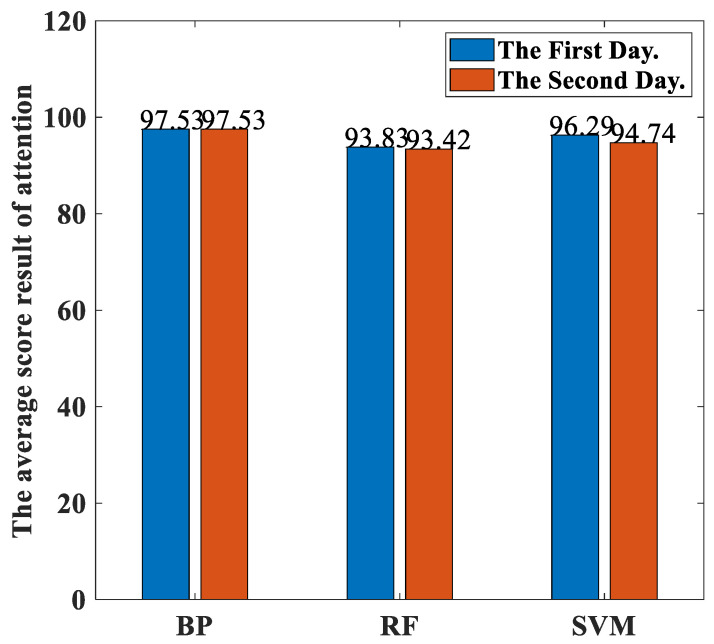
The percentages of the classification accuracies yielded by our proposed method with three different classifiers trained using the EEGs acquired from Hejian performing the reading activity under the concentration state on both the first day and the second day.

Figure 17 shows the percentages of the classification accuracies yielded by our proposed method with three different classifiers trained using the EEGs acquired from Hejian performing the reading activity under the concentration state on both the first day and the second day. It can be seen that the percentages of the classification accuracies trained using the EEGs acquired on the first day are similar to those trained on the second day for all three classifiers. Hence, the dates of the measurements can be ignored for performing the classification.

## 5. Conclusions

The advantage of our proposed method is the ability to perform a subjective and effective attention assessment of the participants when conducting various activities. In particular, this paper proposes a classification method for performing the regression task so that it is not required to have reference values from medical officers. First, FFT is employed for performing denoising. Then, EMD is applied to remove the underlying trend. Next, SSA is employed to increase the total number of components for performing feature extraction. After that, three different types of classifiers, namely the random forest-based classifier, the SVM-based classifier, and the BP neural network-based classifier, are employed for performing the classification. Finally, the percentages of the classification accuracies are employed as the score for concentration or for immersion. The computer numerical simulation results show that the average percentages of the classification accuracies over both 12 participants and over three classifiers yielded by our proposed method are higher than those yielded by the traditional methods for all the activities as well as for both the concentration state and the immersion state. Moreover, it is found that the highest score recorded in the concentration state yielded by our method is 100. This demonstrates that our proposed method yields a better classification performance. In addition, there are similar sorting orders of the activities yielded by both our proposed method and the traditional methods for both the concentration state and the immersion state. This demonstrates that our proposed method is reliable. Furthermore, as it is required to pay more attention when performing the reading activity and the doing computer exercise activity compared to performing the eating activity and the playing toy activity, our obtained results are consistent with this result. Hence, our obtained results are reasonable. Moreover, it is found that it is not necessarily true for the average percentages of the classification accuracies over both these 12 participants and over these three classifiers for performing the various activities yielded by both our proposed method and the traditional methods under the concentration state to be higher than those under the immersion state. Finally, it is found that the random forest-based classifier yields the highest average percentages of the classification accuracies compared to the SVM-based classifier and the BP neural network-based classifier for both the concentration state and the immersion state as well as for both our proposed method and the traditional method.

The limitation of our proposed method is the hardware constraint. Here, only a single-channel EEG device is employed for performing the data acquisition. Hence, the information that can be extracted from the EEGs is limited. To address this issue, more types of signals such as image sequences and motion signals will be used in the future for performing the attention assessment. In particular, a camera and a motion sensor will be employed for acquiring the data. Then, the new set of features extracted from the image sequences and the motion signals will be added to the existing features extracted from the single-channel EEGs. At present, only a small amount of EEGs is acquired. That is why the percentages of the classification accuracies of some cases are 100. In the future, more measurements will be obtained. Hence, the percentages of the classification accuracies will be more reliable.

## Figures and Tables

**Table 1:** The total numbers of the retained samples when different participants are conducting different activities under the concentration state.

Participant	Drawing	Eating	Doing Computer Exercises	Playing Electronic Games	Reading	Playing Toys	Watching Television	Total
Danny	512	41	658	557	548	485	455	3256
Lang	527	214	558	474	468	370	377	2988
Tim	512	76	472	477	546	386	456	2925
Tong	756	292	723	707	764	583	806	4631
Hejian	554	182	571	563	564	537	597	3568
Qunqing	513	260	592	592	574	650	586	3767
Shuyun	596	175	502	586	523	683	600	3665
Xin	271	13	286	212	255	178	288	1503
Yuyuan	506	29	472	551	516	598	612	3284
Aidan	268	154	206	255	219	199	216	1517
Ben	276	163	255	262	304	193	262	1715
Him	88	116	159	109	144	78	91	785
Total	5379	1715	5454	5345	5425	4940	5346	33,604

**Table 2:** The total numbers of the retained samples when different participants are conducting different activities under the immersion state.

Participant	Drawing	Eating	Doing Computer Exercises	Playing Electronic Games	Reading	Playing Toys	Watching Television	Total
Danny	174	25	293	168	121	192	186	1159
Lang	487	80	290	481	433	402	422	2595
Tim	115	171	314	280	220	261	270	1631
Tong	431	262	454	355	313	485	360	2660
Hejian	304	158	417	493	475	455	444	2746
Qunqing	503	275	425	515	470	541	522	3251
Shuyun	584	116	374	568	454	601	397	3094
Xin	291	21	240	263	274	177	158	1424
Yuyuan	370	268	449	540	413	602	482	3124
Aidan	155	228	165	306	213	200	230	1497
Ben	206	84	276	348	260	214	218	1606
Him	187	204	226	211	222	244	200	1494
Total	3807	1892	3923	4528	3868	4374	3889	26,281

**Table 3:** The common human behaviors and the brain waves responsible for these human behaviors.

Waves	Frequency Range	Common Human Behaviors
δ	0.5 Hz–4 Hz	Unconscious level
θ	4 Hz–8 Hz	Creativity and imagination
α	8 Hz–12 Hz	Unconscious before falling asleep
SMR	12 Hz–14.99 Hz	Relaxation and concentration
Mid β	15 Hz–19.99 Hz	Thinking deeply
High β	20 Hz–30 Hz	Excitement and anxiety
γ	30 Hz–49 Hz	Relieving the pressure

**Table 4:** The values of P corresponding to different features extracted from the SSA components of a detrended EEG.

Features	Approximate Entropy	Mean	Interquartile Range	Mean Absolute Deviation	Range	Variance	Skewness	Kurtosis	$L_{1}$ Norm	$L_{2}$ Norm	$L_{\infty }$ Norm
EEG-SSA1	5.97 × 10^−15^	9.06 × 10^−5^	3.50 × 10^−2^	2.93 × 10^−5^	1.28 × 10^−17^	3.65 × 10^−2^	9.66 × 10^−23^	1.06 × 10^−49^	3.65 × 10^−2^	9.17 × 10^−2^	3.65 × 10^−23^
EEG-SSA2	6.49 × 10^−3^	4.51 × 10^−8^	2.75 × 10^−1^	1.43 × 10^−2^	2.73 × 10^−9^	4.98 × 10^−7^	1.41 × 10^−50^	9.94 × 10^−15^	4.99 × 10^−7^	4.53 × 10^−4^	6.54 × 10^−9^
EEG-SSA3	4.75 × 10^−3^	4.11 × 10^−6^	2.64 × 10^−4^	1.12 × 10^−1^	3.71 × 10^−2^	5.41 × 10^−7^	2.04 × 10^−73^	1.47 × 10^−2^	5.41 × 10^−7^	2.47 × 10^−2^	3.46 × 10^−2^
EEG-SSA4	4.49 × 10^−19^	7.14 × 10^−9^	5.73 × 10^−29^	1.35 × 10^−1^	2.69 × 10^−10^	7.02 × 10^−3^	3.21 × 10^−5^	1.28 × 10^−50^	7.03 × 10^−3^	2.24 × 10^−2^	4.27 × 10^−11^
High-β-SSA1	3.71 × 10^−2^	4.60 × 10^−20^	7.91 × 10^−4^	5.73 × 10^−2^	2.54 × 10^−18^	5.92 × 10^−2^	1.93 × 10^−8^	6.60 × 10^−43^	5.92 × 10^−2^	9.22 × 10^−2^	2.13 × 10^−18^
High-β-SSA2	1.65 × 10^−1^	4.60 × 10^−20^	7.02 × 10^−4^	1.31 × 10^−1^	1.27 × 10^−19^	3.49 × 10^−7^	2.52 × 10^−38^	3.38 × 10^−25^	3.48 × 10^−7^	9.57 × 10^−2^	2.47 × 10^−25^
Low-β-SSA1	1.38 × 10^−4^	7.50 × 10^−21^	1.86 × 10^−2^	6.01 × 10^−3^	5.24 × 10^−15^	1.73 × 10^−3^	1.44 × 10^−15^	1.95 × 10^−32^	1.73 × 10^−3^	1.67 × 10^−1^	3.56 × 10^−15^
Low-β-SSA2	1.77 × 10^−8^	7.50 × 10^−21^	3.93 × 10^−2^	2.81 × 10^−3^	4.14 × 10^−16^	1.96 × 10^−14^	1.37 × 10^−33^	4.58 × 10^−15^	1.96 × 10^−14^	6.08 × 10^−2^	6.82 × 10^−20^
Mid-β-SSA1	5.28 × 10^−9^	1.15 × 10^−24^	2.95 × 10^−1^	1.10 × 10^−2^	5.30 × 10^−9^	1.63 × 10^−3^	1.61 × 10^−1^	1.15 × 10^−19^	1.63 × 10^−3^	7.85 × 10^−2^	4.93 × 10^−9^
Mid-β-SSA2	3.61 × 10^−4^	1.15 × 10^−24^	3.99 × 10^−1^	1.46 × 10^−2^	2.87 × 10^−6^	1.54 × 10^−5^	4.13 × 10^−31^	9.97 × 10^−8^	1.54 × 10^−5^	4.44 × 10^−2^	8.17 × 10^−10^
β-SSA1	3.62 × 10^−6^	2.48 × 10^−28^	9.81 × 10^−5^	1.73 × 10^−1^	5.44 × 10^−40^	9.92 × 10^−3^	3.08 × 10^−29^	3.98 × 10^−57^	9.92 × 10^−3^	1.04 × 10^−1^	2.34 × 10^−44^
β-SSA2	3.16 × 10^−1^	2.48 × 10^−28^	1.97 × 10^−4^	1.24 × 10^−1^	1.79 × 10^−5^	1.36 × 10^−2^	3.61 × 10^−24^	1.21 × 10^−8^	1.36 × 10^−2^	1.55 × 10^−1^	4.34 × 10^−9^
γ-SSA1	1.97 × 10^−4^	3.21 × 10^−1^	3.84 × 10^−3^	1.36 × 10^−1^	1.05 × 10^−1^	1.24 × 10^−1^	7.83 × 10^−2^	7.68 × 10^−12^	1.24 × 10^−1^	3.25 × 10^−2^	2.24 × 10^−1^
γ-SSA2	1.22 × 10^−1^	3.21 × 10^−1^	2.46 × 10^−3^	1.44 × 10^−1^	2.07 × 10^−3^	8.36 × 10^−2^	2.12 × 10^−4^	1.60 × 10^−8^	8.36 × 10^−2^	4.04 × 10^−2^	3.64 × 10^−4^
ɑ-SSA1	1.77 × 10^−16^	3.23 × 10^−1^	2.94 × 10^−2^	1.60 × 10^−9^	1.67 × 10^−5^	2.89 × 10^−4^	2.03 × 10^−6^	2.66 × 10^−4^	2.89 × 10^−4^	1.31 × 10^−3^	1.87 × 10^−5^
ɑ-SSA2	3.54 × 10^−6^	3.23 × 10^−1^	1.49 × 10^−2^	3.41 × 10^−11^	2.04 × 10^−14^	9.81 × 10^−16^	4.32 × 10^−3^	1.67 × 10^−8^	9.81 × 10^−16^	4.59 × 10^−6^	6.23 × 10^−14^
θ-SSA1	6.45 × 10^−3^	8.77 × 10^−7^	9.28 × 10^−3^	3.81 × 10^−9^	6.34 × 10^−3^	4.77 × 10^−3^	3.46 × 10^−3^	1.44 × 10^−1^	4.77 × 10^−3^	5.23 × 10^−3^	5.36 × 10^−3^
θ-SSA2	5.64 × 10^−3^	8.77 × 10^−7^	1.35 × 10^−1^	3.79 × 10^−4^	1.82 × 10^−4^	1.09 × 10^−3^	2.46 × 10^−11^	9.33 × 10^−5^	1.09 × 10^−3^	7.74 × 10^−2^	3.27 × 10^−5^
δ-SSA1	1.67 × 10^−17^	1.84 × 10^−1^	8.43 × 10^−28^	1.96 × 10^−24^	3.24 × 10^−24^	1.41 × 10^−26^	4.86 × 10^−2^	9.59 × 10^−2^	1.41 × 10^−26^	1.97 × 10^−27^	2.83 × 10^−24^
δ-SSA2	1.45 × 10^−8^	1.84 × 10^−1^	5.90 × 10^−17^	8.29 × 10^−21^	4.98 × 10^−13^	2.84 × 10^−16^	1.06 × 10^−1^	6.84 × 10^−2^	2.84 × 10^−16^	1.55 × 10^−18^	1.01 × 10^−13^
SMR-SSA1	4.43 × 10^−10^	5.30 × 10^−5^	1.73 × 10^−1^	1.21 × 10^−4^	6.37 × 10^−5^	1.97 × 10^−3^	3.93 × 10^−11^	1.11 × 10^−5^	1.97 × 10^−3^	9.44 × 10^−3^	5.83 × 10^−5^
SMR-SSA2	2.57 × 10^−4^	5.30 × 10^−5^	2.29 × 10^−1^	5.13 × 10^−5^	8.28 × 10^−13^	2.01 × 10^−12^	9.11 × 10^−9^	6.09 × 10^−11^	2.01 × 10^−12^	2.27 × 10^−3^	9.75 × 10^−13^

**Table 5:** The percentages of the classification accuracies for 12 participants performing the various activities under the concentration state yielded by the traditional methods.

Participant	Classifier	Drawing	Eating	Doing Computer Exercises	Playing Electronic Games	Reading	Playing Toys	Watching Television
Danny	BP	85.53	50.00	72.22	85.62	77.48	58.05	87.5
	RF	84.91	37.50	84.09	83.66	82.88	59.77	92.86
	SVM	83.65	58.30	74.62	79.74	77.48	63.79	88.10
Lang	BP	83.22	80.56	88.89	75.53	91.80	82.28	77.48
	RF	87.07	77.78	88.51	87.88	90.51	79.28	86.55
	SVM	89.12	79.16	87.36	86.71	93.59	79.28	80.70
Tong	BP	77.44	76.79	91.48	78.51	90.78	73.06	88.69
	RF	89.23	78.06	93.43	90.03	89.01	83.34	90.52
	SVM	85.13	75.10	90.51	88.16	91.85	82.70	85.63
Hejian	BP	80.07	47.22	86.77	81.76	88.58	63.50	86.57
	RF	85.14	63.89	88.10	93.69	90.68	54.98	88.56
	SVM	80.43	61.81	88.36	89.19	93.01	49.88	87.81
Shuyun	BP	68.75	60.95	58.41	66.47	87.59	56.91	68.61
	RF	67.24	58.09	84.96	72.71	87.35	55.99	78.06
	SVM	65.34	59.05	82.89	71.54	86.86	57.09	74.72
Tim	BP	66.67	71.01	88.77	95.24	81.31	67.93	77.36
	RF	66.67	76.81	92.98	95.64	88.89	72.57	86.83
	SVM	70.48	72.47	92.98	96.83	84.34	75.11	88.48
Qunqing	BP	51.21	78.27	76.82	66.45	80.38	68.71	80.04
	RF	61.37	93.59	90.36	76.77	87.71	76.28	87.90
	SVM	66.00	91.88	86.98	74.41	87.00	73.41	87.90
Xin	BP	65.04	66.67	81.02	57.81	56.28	76.54	73.61
	RF	74.39	75.00	80.09	77.61	69.26	76.54	70.83
	SVM	75.61	58.33	77.31	70.83	70.99	79.07	63.89
Yuyuan	BP	86.19	62.96	66.91	78.40	62.90	75.37	80.46
	RF	82.89	66.67	77.53	87.45	70.97	83.34	74.25
	SVM	81.38	66.67	75.56	86.62	71.50	75.92	72.41
Aidan	BP	56.92	72.16	66.67	82.09	64.53	63.8	82.09
	RF	69.23	82.78	73.16	82.71	72.34	71.903	82.71
	SVM	66.66	82.41	72.72	80.42	61.7	77.62	80.42
Ben	BP	75.76	52.38	70.46	63.63	83.49	53.28	63.63
	RF	77.88	63.64	78.06	78.54	88.89	55.74	78.54
	SVM	76.67	69.26	74.68	76.77	87.3	52.19	76.77
Him	BP	63.09	56.25	89.9	79.01	86.67	75.76	79.01
	RF	69.05	57.64	95.96	74.07	98.09	76.52	74.07
	SVM	59.52	69.45	91.92	79..01	91.43	75.76	79..01
	Mean:	74.30	68.07	82.26	80.35	82.37	69.53	80.67

**Table 6:** The percentages of the classification accuracies for 12 participants performing the various activities under the immersion state via yielded by the traditional methods.

Participant	Classifier	Drawing	Eating	Doing Computer Exercises	Playing Electronic Games	Reading	Playing Toys	Watching Television
Danny	BP	72.33	70.83	72.35	81.04	84.68	53.45	82.14
	RF	82.39	62.5	76.52	92.81	90.09	59.77	91.6
	SVM	89.31	62.5	77.65	84.97	84.69	63.22	92.26
Lang	BP	84.81	65.28	85.44	76.92	84.61	66.37	76.03
	RF	92.29	59.72	88.51	84.38	89.23	87.39	79.24
	SVM	91.84	72.22	91.57	86.71	95.13	88.89	81.58
Tong	BP	77.69	83.97	86.86	89.10	89.36	70.32	81.35
	RF	77.43	84.81	83.70	86.92	92.20	74.20	80.43
	SVM	78.98	84.81	84.91	90.96	92.91	78.54	82.88
Hejian	BP	76.81	61.80	77.77	83.33	93.24	48.90	87.31
	RF	84.78	63.89	83.33	88.51	92.54	65.69	91.29
	SVM	83.33	56.25	87.30	87.84	91.84	65.45	86.82
Shuyun	BP	63.64	63.81	66.37	58.48	88.32	48.06	73.89
	RF	70.27	63.81	74.63	71.73	94.89	63.35	76.39
	SVM	69.32	65.80	77.58	69.40	92.21	58.20	77.22
Tim	BP	66.67	76.81	87.37	94.45	80.30	66.67	80.66
	RF	62.86	71.02	90.18	96.83	90.91	76.79	88.89
	SVM	61.90	73.91	87.02	96.43	91.41	79.32	88.48
Qunqing	BP	71.30	85.90	85.94	78.49	85.11	75.05	87.05
	RF	76.38	80.77	83.59	81.72	88.41	82.82	88.32
	SVM	67.77	86.33	86.46	78.06	86.29	79.75	88.32
Xin	BP	63.41	75.00	69.45	65.62	52.38	70.37	59.03
	RF	72.36	91.67	76.85	71.87	72.30	71.60	73.61
	SVM	73.98	58.33	75.93	75.52	74.89	70.37	65.27
Yuyuan	BP	74.77	44.45	75.06	85.39	72.31	72.41	76.32
	RF	81.08	74.08	74.57	81.27	81.72	70.37	77.47
	SVM	76.28	77.78	74.81	84.57	77.96	71.85	76.55
Aidan	BP	56.12	70.67	90	74.11	71.63	69.79	61.72
	RF	65.4	68	96.67	60.06	82.27	73.96	64.58
	SVM	67.93	73.33	95	61.16	77.31	79.69	67.71
Ben	BP	74.24	71.77	45.99	74.35	70.97	55.55	66.87
	RF	86.36	79.93	79.32	83.33	89.25	56.41	77.57
	SVM	81.31	70.41	77.63	87.18	82.79	50.85	71.92
Him	BP	60.33	48.11	72.84	76.77	84.66	55.19	90.61
	RF	63.98	67.83	77.95	85.25	86.24	87.7	93.08
	SVM	68.85	63.19	79.89	86.34	83.86	80.33	95.55
	Mean:	74.12	70.31	80.47	80.88	84.41	69.12	80.00

**Table 7:** The percentages of the classification accuracies for 12 participants performing the various activities under the concentration state yielded by our method.

Participant	Classifier	Drawing	Eating	Doing Computer Exercises	Playing Electronic Games	Reading	Playing Toys	Watching Television
Danny	BP	89.94	76.39	86.53	90.07	92.15	56.70	88.92
	RF	94.37	41.67	89.84	92.73	93.84	59.45	98.15
	SVM	89.94	80.56	93.6	90.23	92.25	48.15	89.43
Lang	BP	94.65	80.17	95.12	96.5	95.35	87.39	80.23
	RF	93.40	72.57	97.00	97.67	96.77	93.99	91.52
	SVM	95.01	58.01	89.77	96.27	91.11	84.68	86.84
Tong	BP	91.80	77.06	95.49	83.95	90.31	84.98	92.07
	RF	93.34	90.52	95.16	96.37	92.17	90.57	97.72
	SVM	94.43	90.52	94.66	93.97	87.40	94.12	92.19
Hejian	BP	85.77	55.55	88.69	88.07	95.11	57.84	80.14
	RF	95.81	55.09	96.07	91.13	95.55	57.84	91.29
	SVM	76.71	63.43	89.4	82.57	86.21	68.65	78.63
Shuyun	BP	82.80	57.45	77.32	80.77	80.10	56.02	82.35
	RF	86.17	48.23	85.75	81.83	90.13	61.35	87.62
	SVM	81.03	48.93	78.56	72.66	80.90	64.26	80.60
Tim	BP	74.23	75.36	95.92	97.15	94.64	77.29	87.14
	RF	80.63	89.85	97.50	95.14	95.75	74.51	87.64
	SVM	76.01	91.30	99.74	91.46	87.67	84.31	89.64
Qunqing	BP	80.75	87.61	91.87	78.16	85.72	71.20	90.80
	RF	86.13	95.72	97.18	74.57	91.84	76.47	91.56
	SVM	71.43	89.32	92.77	73.42	82.04	75.12	89.90
Xin	BP	71.14	83.33	74.86	80.21	76.19	73.33	83.52
	RF	82.93	25.00	83.62	88.02	80.08	91.52	91.01
	SVM	64.64	16.67	78.25	70.84	64.07	86.67	79.96
Yuyuan	BP	87.45	66.67	79.11	86.71	79.15	70.37	76.73
	RF	88.12	66.67	80.80	93.64	86.05	82.78	87.76
	SVM	82.46	40.74	78.48	83.62	71.37	74.82	76.37
Aidan	BP	53.9	77.14	85.84	84.85	95.56	55.39	77.29
	RF	72.34	83.81	95.21	86.58	94.44	76.41	97.07
	SVM	49.64	78.57	82.09	50.22	86.11	55.9	69.23
Ben	BP	80.64	65.03	83.84	84.81	92.53	90.3	82.25
	RF	89.84	61.75	85.86	86.92	85.63	83.63	77.06
	SVM	98.41	47.54	86.87	92.41	86.21	70.91	80.08
Him	BP	85.72	93.18	85.18	94.95	90.28	89.28	93.06
	RF	94.29	77.27	96.3	95.96	90.28	89.29	97.22
	SVM	96.19	62.12	85.19	80.81	81.94	57.14	95.14
	Mean:	83.66	68.63	88.60	86.26	88.75	74.24	86.61

**Table 8:** The percentages of the classification accuracies for 12 participants performing the various activities under the immersion state via yielded by our method.

Participant	Classifier	Drawing	Eating	Doing Computer Exercises	Playing Electronic Games	Reading	Playing Toys	Watching Television
Danny	BP	93.08	50.00	77.65	92.81	93.29	63.79	97.62
	RF	97.49	62.50	87.50	100	98.89	80.46	95.24
	SVM	96.22	12.50	76.14	94.77	96.56	75.86	98.81
Lang	BP	96.37	80.55	96.55	96.67	97.18	90.44	78.06
	RF	97.05	97.22	99.62	97.11	98.72	94.17	83.65
	SVM	97.51	100	100	98.00	99.23	99.07	81.13
Tong	BP	88.21	82.70	92.46	92.21	87.94	88.81	88.99
	RF	93.85	86.92	92.70	93.46	95.04	90.87	95.11
	SVM	82.82	90.72	95.13	92.53	98.58	81.51	89.60
Hejian	BP	87.68	63.89	88.09	86.26	96.97	55.23	87.56
	RF	88.77	59.72	92.59	90.99	96.97	74.21	91.05
	SVM	93.48	63.89	91.27	92.79	98.13	60.82	91.05
Shuyun	BP	86.18	63.81	81.12	76.02	87.59	60.77	77.78
	RF	90.53	77.14	82.01	80.31	97.57	63.54	84.17
	SVM	88.63	62.86	86.73	85.77	97.81	60.96	88.06
Tim	BP	80.00	77.68	94.39	95.24	92.93	70.04	94.24
	RF	88.57	80.79	94.74	95.24	98.99	86.92	95.48
	SVM	93.33	65.25	85.96	96.82	95.45	65.82	87.24
Qunqing	BP	79.47	85.66	91.66	68.39	83.22	82.00	89.81
	RF	90.73	88.89	95.31	81.51	89.83	85.89	92.99
	SVM	88.96	88.89	86.20	80.21	92.20	82.41	92.57
Xin	BP	68.96	66.67	77.78	77.97	68.06	81.50	68.75
	RF	82.02	72.22	88.43	85.80	90.28	79.63	78.47
	SVM	88.89	52.78	90.28	87.25	89.93	75.93	84.72
Yuyuan	BP	81.98	63.60	78.19	84.16	80.58	73.01	75.17
	RF	87.39	70.97	87.90	89.71	88.98	70.65	88.74
	SVM	91.89	67.61	88.64	91.77	93.82	81.52	92.41
Aidan	BP	83.33	59.23	94.53	78.38	80.85	82.81	72.15
	RF	89.33	69.69	96.17	85.16	91.49	89.58	72.15
	SVM	76	80.72	98.36	84.90	87.94	78.13	75.95
Ben	BP	75.51	83.33	89.45	84.04	83.87	57.26	89.39
	RF	82.99	87.18	89.45	90.10	93.01	66.67	91.92
	SVM	69.39	47.44	73	74.55	82.26	64.96	90.40
Him	BP	93.91	82.11	72.11	90.62	90.74	83.33	82.72
	RF	91.88	88.34	83.51	90.12	95.24	85.52	88.56
	SVM	68.11	70.46	74.21	87.66	79.37	86.88	93.19
	Mean:	86.96	72.33	88.05	88.04	91.34	76.97	86.80

**Table 9:** The percentages of the classification accuracies yielded by our proposed method via the various classifiers based on the EEGs at different durations of the samples acquired from Hejian performing the reading activity under the concentration state.

Classifier	1 s	3 s	5 s	10 s
BP	96.98	97.44	97.83	95.67
RF	99.57	96.15	97.83	97.26
SVM	86.21	85.46	82.61	83.33

**Table 10:** The total times required for performing the attention assessments for each individual for performing each activity under both the concentration state and the immersion state via the traditional methods with all three classifiers.

Participant	Drawing	Eating	Doing Computer Exercises	Playing Electronic Games	Reading	Playing Toys	Watching Television
Danny	709.70 s	71.95 s	927.71 s	697.34 s	641.60 s	627.36 s	635.24 s
Lang	940.26 s	275.26 s	769.41 s	894.02 s	831.76 s	709.91 s	742.84 s
Tong	1062.41 s	515.44 s	1069.46 s	932.55 s	942.4 s	952.05 s	1031.80 s
Hejian	761.28 s	312.02 s	874.18 s	927.25 s	902.91 s	883.16 s	926.26 s
Shuyun	1072.72 s	270.45 s	774.54 s	1030.56 s	868.76 s	1146.33 s	895.83 s
tim	548.15 s	227.59 s	698.00 s	655.50 s	692.59 s	583.30 s	641.51 s
Qunqing	924.83 s	488.12 s	894.13 s	981.36 s	922.29 s	1057.49 s	977.78 s
Xin	516.40 s	36.64 s	469.02 s	425.69 s	475.37 s	231.21 s	402.45 s
Yuyuan	824.14 s	277.28 s	842.77 s	977.13 s	835.07 s	1088.47 s	981.35 s
Aidan	393.87 s	357.31 s	363.05 s	515.95 s	402.47 s	359.22 s	422.09 s
Ben	444.68 s	225.48 s	505.39 s	555.14 s	538.93 s	373.06 s	450.18 s
Him	262.02 s	298.34 s	357.24 s	283.56 s	347.62 s	296.94 s	263.68 s
Total	54,318.57 s						

**Table 11:** The total times required for performing the attention assessments for each individual for performing each activity under both the concentration state and the immersion state via our proposed method with all three classifiers.

Participant	Drawing	Eating	Doing Computer Exercises	Playing Electronic Games	Reading	Playing Toys	Watching Television
Danny	1148.42 s	123.19 s	1710.73 s	1185.66 s	1115.83 s	1137.53 s	1080.97 s
Lang	1747.66 s	456.14 s	1421.33 s	1618.29 s	1498.82 s	1256.50 s	1315.50 s
Tong	1845.90 s	853.62 s	1943.68 s	1720.52 s	1742.65 s	1733.83 s	1906.92 s
Hejian	1331.78 s	552.95 s	1597.31 s	1737.92 s	1682.20 s	1589.75 s	1691.95 s
Shuyun	1873.23 s	538.36 s	1319.43 s	1831.15 s	1508.13 s	2066.58 s	1464.77 s
tim	906.35 s	386.56 s	1188.31 s	1145.20 s	1160.97 s	947.82 s	1092.37 s
Qunqing	1515.19 s	851.28 s	1606.93 s	1769.88 s	1655.20 s	1818.04 s	1772.01 s
Xin	900.74 s	60.48 s	835.49 s	744.60 s	843.07 s	432.20 s	794.56 s
Yuyuan	1596.38 s	462.60 s	1495.88 s	1868.94 s	1572.73 s	1905.77 s	1876.69 s
Aidan	661.08 s	699.39 s	694.03 s	944.60 s	744.41 s	677.35 s	787.90 s
Ben	772.15 s	387.60 s	909.92 s	899.57 s	913.85 s	628.86 s	808.83 s
Him	465.96 s	506.74 s	640.81 s	488.96 s	590.09 s	522.33 s	504.93 s
Total	96,804.8 s						

**Table 12:** The optimal parameters in the SVM in our proposed method trained using the EEGs acquired from Hejian performing the reading activity under the concentration state on both the first day and the second day.

Date	Value of the Box Constraint	Type of the Kernel Functions	Parameter in the Kernel Function: Polynomial Order or Kernel Scale
First day	23.891	Polynomial	2
Second day	127.57	Gaussian	140.88

## Data Availability

The data will be provided based on the request manner.

## References

[1] Mohammadpour M., Mozaffari S. Classification of EEG-Based Attention for Brain Computer Interface. Proceedings of the 2017 3rd lranian Conference on Intelligent Systems and Signal Processing.

[2] Pai W., Mei W., Fan W., Xue-Bin Q. Research on Attention Classification Based on Long Short-term Memory Network. Proceedings of the 2020 5th International Conference on Mechanical, Control and Computer Engineering.

[3] Teplan M. (2002). Fundamentals of EEG measurement. Meas. Sci. Rev..

[4] Ray W.J., Cole H.W. (1985). EEG alpha activity reflects attentional demands and beta activity reflects emotional and cognitive processes. Science.

[5] Schacter D.L. (1977). EEG theta waves and psychological phenomena: A review and analysis. Biol. Psychol..

[6] Harmony T., Fernández T., Silva J., Bernal J., Díaz-Comas L., Reyes A., Marosi E., Rodriguez M., Rodriguez M. (1996). EEG delta activity: An indicator of attention to internal processing during performance of mental tasks. Int. J. Psychophysiol..

[7] Wolpaw J.R., Birbaumer N., McFarland D.J., Pfurtscheller G., Vaughan T.M. (2002). Brain–computer interfaces for communication and control. Clin. Neurophysiol..

[8] Zhao G., Ge Y., Shen B., Wei X., Wang H. (2018). Emotion Analysis for Personality Inference from EEG Signals. IEEE Trans. Affect. Comput..

[9] Huang Z., Ling B.W.-K. (2002). Sleeping stage classification based on joint quaternion valued singular spectrum analysis and ensemble empirical mode decomposition. Biomed. Signal Process. Control.

[10] Delisle-Rodriguez D., Villa-Parra A.C., Bastos-Filho T., López-Delis A., Frizera-Neto A., Krishnan S., Rocon E. (2017). Adaptive Spatial Filter Based on Similarity Indices to Preserve the Neural Information on EEG Signals during On-Line Processing. Sensors.

[11] Liu Y., Li M., Zhang H., Wang H., Li J., Jia J., Wu Y., Zhang L. (2014). A tensor-based scheme for stroke patients’ motor imagery EEG analysis in BCI-FES rehabilitation training. J. Neurosci. Methods.

[12] Shenjie S., Thomas K.P., Smitha K.G., Vinod A.P. Two player EEG-based neurofeedback ball game for attention enhancement. Proceedings of the 2014 IEEE International Conference on Systems, Man, and Cybernetics (SMC).

[13] Khong A., Jiangnan L., Thomas K., Vinod A.P. BCI based multi-player 3-D game control using EEG for enhancing attention and memory. Proceedings of the 2014 IEEE International Conference on Systems, Man, and Cybernetics (SMC).

[14] Mohammadi S., Enshaeifar S., Ghavami M., Sanei S. Classification of awake, REM and NREM from EEG via singular spectrum analysis. Proceedings of the 2015 37th Annual International Conference of the IEEE Engineering in Medicine and Biology Society.

[15] Mohammadi S., Kouchaki S., Ghavami M., Sanei S. (2016). Improving time-frequency domain sleep EEG classification via singular spectrum analysis. J. Neurosci. Methods.

[16] Maddirala A., Shaik R. (2016). Motion artifact removal from single channel electroencephalogram signals using singular spectrum analysis. Biomed. Signal Process. Control.

[17] Maddirala A., Shaik R. (2016). Removal of EOG Artifacts from single channel EEG signals using combined singular spectrum analysis and adaptive noise canceler. IEEE Sens. J..

[18] Ayain S., Saraoglu H., Kara S. (2011). Singular Spectrum Analysis of Sleep EEG in Insomnia. J. Med. Syst..

[19] Golyandina N., Nekrutkin V., Zhigljavsky A.A. (2001). Analysis of Time Series Structure: SSA and Related Techniques.

[20] Hassani H. (2007). Singular spectrum analysis: Methodology and comparison. J. Data Sci..

[21] Vijayasankar A., Kumar P.R. Correction of blink artifacts from single channel EEG by EMD-IMF thresholding. Proceedings of the 2018 Conference on Signal Processing and Communication Engineering Systems (SPACES).

[22] Khare S.K., Gaikwad N.B., Bajaj V. (2022). VHERS: A Novel Variational Mode Decomposition and Hilbert Transform-Based EEG Rhythm Separation for Automatic ADHD Detection. IEEE Trans. Instrum. Meas..

[23] Mohguen W., El’hadi Bekka R. Comparative Study of ECG Signal Denoising by Empirical Mode Decomposition and Thresholding Functions. Proceedings of the 2019 6th International Conference on Electrical and Electronics Engineering (ICEEE).

[24] Lin P., Kuang W., Liu Y., Ling B.W.-K. (2019). Grouping and selecting singular spectrum analysis components for denoising via empirical mode decomposition approach. Circuits Syst. Signal Process..

[25] Srinivasan V., Eswaran C., Sriraam N. (2007). Approximate Entropy-Based Epileptic EEG Detection Using Artificial Neural Networks. IEEE Trans. Inf. Technol. Biomed..

[26] Hu L., Zhao K., Zhou X., Ling B.W.-K., Liao G. (2020). Empirical Mode Decomposition Based Multi-Modal Activity Recognition. Sensors.

[27] Li H. (2019). Network traffic prediction of the optimized BP neural network based on Glowworm Swarm Algorithm. Syst. Sci. Control. Eng..

[28] Yang X., Yu Q., He L., Guo T. (2013). The one-against-all partition based binary tree support vector machine algorithms for multi-class classification. Neurocomputing.

[29] Minasyan G.R., Chatten J.B., Harner R.N. Detection of epileptiform activity in unresponsive patients using ANN. Proceedings of the 2009 International Joint Conference on Neural Networks.

[30] AIa K., Byeon J.G., Timashev S.F., Vstovskiĭ G.V., Park B.W. (2006). Functional variability of the autocorrelation structure of the EEG. Zhurnal Vyss. Nervn. Deiatelnosti Im. IP Pavlov..

